# Phylogenetic and Functional Analysis of Metagenome Sequence from High-Temperature Archaeal Habitats Demonstrate Linkages between Metabolic Potential and Geochemistry

**DOI:** 10.3389/fmicb.2013.00095

**Published:** 2013-05-15

**Authors:** William P. Inskeep, Zackary J. Jay, Markus J. Herrgard, Mark A. Kozubal, Douglas B. Rusch, Susannah G. Tringe, Richard E. Macur, Ryan deM. Jennings, Eric S. Boyd, John R. Spear, Francisco F. Roberto

**Affiliations:** ^1^Department of Land Resources and Environmental Sciences, Montana State UniversityBozeman, MT, USA; ^2^Thermal Biology Institute, Montana State UniversityBozeman, MT, USA; ^3^Novo Nordisk Foundation Center for Biosustainability, Technical University of DenmarkHørsholm, Denmark; ^4^Center for Genomics and Bioinformatics, Indiana UniversityBloomington, IN, USA; ^5^Department of Energy, Joint Genome InstituteWalnut Creek, CA, USA; ^6^Department of Chemistry and Biochemistry, Montana State UniversityBozeman, MT, USA; ^7^Department of Civil and Environmental Engineering, Colorado School of MinesGolden, CO, USA; ^8^Newmont Mining CorporationEnglewood, CO, USA

**Keywords:** archaea, thermophilic archaea and bacteria, geochemistry, phylogeny, functional genomics

## Abstract

Geothermal habitats in Yellowstone National Park (YNP) provide an unparalleled opportunity to understand the environmental factors that control the distribution of archaea in thermal habitats. Here we describe, analyze, and synthesize metagenomic and geochemical data collected from seven high-temperature sites that contain microbial communities dominated by archaea relative to bacteria. The specific objectives of the study were to use metagenome sequencing to determine the structure and functional capacity of thermophilic archaeal-dominated microbial communities across a pH range from 2.5 to 6.4 and to discuss specific examples where the metabolic potential correlated with measured environmental parameters and geochemical processes occurring *in situ*. Random shotgun metagenome sequence (∼40–45 Mb Sanger sequencing per site) was obtained from environmental DNA extracted from high-temperature sediments and/or microbial mats and subjected to numerous phylogenetic and functional analyses. Analysis of individual sequences (e.g., MEGAN and G + C content) and assemblies from each habitat type revealed the presence of dominant archaeal populations in all environments, 10 of whose genomes were largely reconstructed from the sequence data. Analysis of protein family occurrence, particularly of those involved in energy conservation, electron transport, and autotrophic metabolism, revealed significant differences in metabolic strategies across sites consistent with differences in major geochemical attributes (e.g., sulfide, oxygen, pH). These observations provide an ecological basis for understanding the distribution of indigenous archaeal lineages across high-temperature systems of YNP.

## Introduction

*Archaea* are now recognized as the third domain of Life and are considered an ancestral link to the Eukarya (Woese and Fox, 1977; Woese et al., 1990). Although early research on organisms within this domain often focused on extreme thermophilic or halophilic organisms (Stetter, 2006), it is now established that archaea are not limited to extreme environments, and recent findings demonstrate the broad distribution of members of this domain across a wide range of environments including soil, human, marine, and aquatic habitats (Chaban et al., 2006; Auguet et al., 2009; Pester et al., 2011). The role of archaea in contemporary and past environments has been the subject of considerable debate, and members of this group have now been implicated as “missing links” in major global element cycling, including methanogenesis and nitrification in marine systems (DeLong, 2005; Schleper et al., 2005; Falkowski et al., 2008). The evolutionary history of these organisms has been defined in part by different environmental contexts involving variations in oxygen, iron, sulfur, and/or other major crustal or atmospheric components (e.g., CO_2_, CH_4_, H_2_, NH_4_). Metagenome sequencing of well-characterized, high-temperature geothermal systems with variable geochemistry provides a unique opportunity for understanding the metabolic attributes important to specific taxa within the *Archaea* (Inskeep et al., 2010). Contemporary thermophilic archaea in Yellowstone National Park (YNP) occupy a wide range of habitats with regard to dissolved oxygen, sulfide, iron, hydrogen, and pH (e.g., Inskeep et al., 2005; Meyer-Dombard et al., 2005). Consequently, the field environments of YNP provide a natural laboratory where a subset of geochemical parameters varies across different springs, and selects for different types of microbial communities dominated by archaea.

Our understanding of archaeal diversity in Yellowstone hinges primarily on cultivation-based studies, although the past decade has seen an increase in genetic-based investigations. Much early interest centered on members of the order Sulfolobales (phylum Crenarchaeota), and several of these acidophiles were cultivated as the first recognized archaea (Brock et al., 1972; Brierley and Brierley, 1973). These organisms are distributed globally in hydrothermal vents or solfataras and have generally been cultivated at low pH (1–3) using reduced forms of S, Fe and complex C as electron donors under aerobic to microaerobic conditions. The oxidation of Fe(II) is less studied in the Sulfolobales, however, recent work in YNP shows that *Metallosphaera yellowstonensis* populations occupy acidic Fe(III)-oxide mats and contain genes required to oxidize Fe(II) via a *fox* terminal oxidase complex (Kozubal et al., 2008, 2011). The distribution of members of the orders Desulfurococcales and Thermoproteales has not been studied with great detail in YNP, however, these organisms have generally been observed in sulfidic sediments and in higher pH systems (pH 3–8) compared to the Sulfolobales (Barnes et al., 1994; Inskeep et al., 2005; Meyer-Dombard et al., 2005). Two acidophilic Desulfurococcales were isolated from hypoxic sulfur sediments in Norris Geyser Basin (YNP) as obligate anaerobes growing on complex carbon sources and elemental S as an electron acceptor (Boyd et al., 2007). In addition, we have recently obtained a *Pyrobaculum*-like isolate from YNP that also grows with elemental sulfur and complex carbon sources (Macur et al., 2013). However, the distribution of different types of Thermoproteales in YNP and their role in community function is not known. Other less understood members of the domain *Archaea* also occur in YNP geothermal systems, and include members of the Euryarchaeota (e.g., Segerer et al., 1988), Korarchaeota (Elkins et al., 2008; Miller-Coleman et al., 2012), Nanoarchaeota (Clingenpeel et al., 2011), and Thaumarchaeota (Brochier-Armanet et al., 2008; de la Torre et al., 2008; Hatzenpichler et al., 2008; Spang et al., 2010; Beam et al., 2011; Pester et al., 2011). Factors responsible for the distribution of these and other novel phyla to be discussed herein have not been determined, and in many cases, cultivated relatives of these organisms do not exist to inform on their physiology or function *in situ*.

Seven high-temperature geothermal systems were sampled to represent a range of geochemical conditions and to investigate effects of pH, dissolved gases (sulfide, oxygen, hydrogen), and Fe on the distribution and function of archaea in YNP. The primary objectives of this study were to (i) determine the community structure and function of high-temperature archaeal-dominated microbial communities across a pH range from 2.5 to 6.4 utilizing metagenome sequencing, (ii) compare inferred functional attributes across sites and different phylotypes using assembled metagenome sequence to obtain protein family abundances and functional gene content, and (iii) evaluate environmental parameters and geochemical processes across sites that may define the distribution patterns of thermophilic archaea in YNP.

## Results

### Geochemical context and element cycling

**Figure 1:**
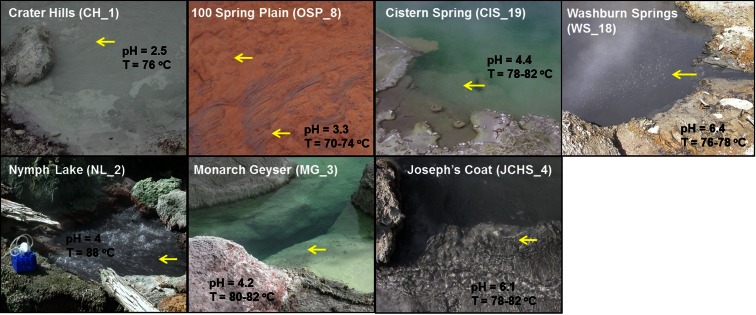
**Site photographs of high-temperature chemotrophic systems in Yellowstone National Park (YNP) selected for metagenome sequencing and described in the current study**. The sites cover a range in geochemical conditions including sulfur-rich sediments from pH 2.5 (CH_1) to 6.4 (WS_18) and higher oxygen environments where the oxidation of ferrous-iron results in the formation of Fe(III)-oxide microbial mats (OSP_8) [pH and temperature measured on site; yellow arrows indicate sampling locations; all site locations referenced in Table 1].

The thermal (70–85°C) sites discussed here range in pH from 2.5 to 6.4 (Table 1), and represent several of the major chemotrophic habitat types observed in Yellowstone’s geothermal basin. Six of the communities are hypoxic and contain elemental sulfur (and/or dissolved sulfide). Consequently, there is significant potential for chemotrophic metabolism based on sulfur oxidation-reduction reactions in these habitats (e.g., Amend and Shock, 2001; Inskeep et al., 2005). The acidic *Crater Hills* (CH_1) and *Nymph Lake* (NL_2) sites are highly turbulent pools that contain suspended solids of elemental sulfur and SiO_2_, and only low levels of dissolved sulfide (e.g., <5 μM) (Figure 1). Metagenome sequence was also obtained from four mildly acidic, sulfidic sediments at *Monarch Geyser* (MG_3), *Cistern Spring* (CIS_19), *Joseph’s Coat Hot Springs* (JCHS_4), and *Washburn Springs* (WS_18) (Table 1). The presence of dissolved sulfide results in the deposition of elemental sulfur in all of these sites (Xu et al., 1998, 2000; Macur et al., 2013); however, pyrite and amorphous Fe-sulfides are important solid phases at JCHS_4 and WS_18, and significant [>1% (w/w)] amounts of stibnite (Sb_2_S_3_) and orpiment (As_2_S_3_) are also present in sediments from JCHS_4 (Table 1; Figure 1). Metagenome analysis of a similar JCHS_4 sediment sample obtained 1 year prior to the current study (Inskeep et al., 2010) showed a community dominated by two different Thermoproteales populations and one member of the order Desulfurococcales. Prior 16S rRNA gene surveys have also suggested that crenarchaeal populations similar to those observed in JCHS are important in the sulfur sediments at CIS_19 and MG_3 (Macur et al., 2013). The predominant dissolved ions in the highly sulfidic (hypoxic) system at *Washburn Springs* (WS_18) are ammonium and sulfate (∼24 mM NH_4_, 17 mM sulfate), although high levels of dissolved hydrogen (450 nM), methane (14.9 μM), inorganic carbon (DIC) (5.5 mM), and organic carbon (DOC) (0.3 mM) were also measured in this study (Table 1; Table S2 in Supplementary Material, Inskeep et al., 2013).

**Table 1:** **Sample location, total dissolved (<0.2 μm) geochemical parameters, and predominant solid phases associated with high-temperature, archaeal-dominated microbial communities in Yellowstone National Park (YNP)**.

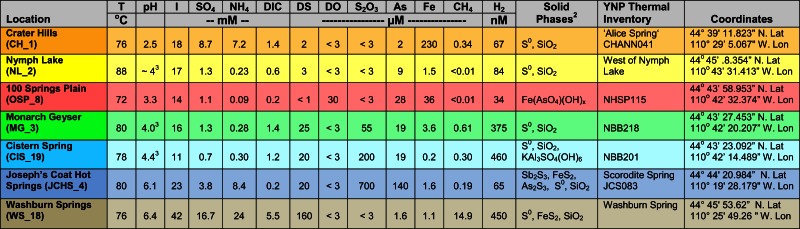

*I, ionic strength calculated from aqueous geochemical modeling at sample temperature; DIC, dissolved inorganic C; DS, dissolved sulfide; S_2_O_3_^2^, thiosulfate; DO, dissolved oxygen*.

*^1^Predominant solid phases determined using scanning electron microscopy (FE-SEM) coupled with energy dispersive analysis of X-rays (EDAX) and X-ray diffraction (XRD)*.

*^2^pH values at these sites have been noted to vary (MG_3; pH ∼ 4–4.5; CIS_19; pH ∼ 4.4–4.9). Also, see USGS reports (e.g., Ball et al., 1998, 2002; McCleskey et al., 2005)*.

The oxidation of dissolved sulfide with oxygen occurs via abiotic and/or biotic processes (Xu et al., 1998; Friedrich et al., 2005; Ghosh and Dam, 2009), and results in the deposition of elemental sulfur commonly observed within sulfidic systems of YNP (Figure 1). Moreover, the products of sulfide oxidation are pH dependent and vary due to kinetic favorability of specific reaction steps (Xu et al., 1998, 2000). Thiosulfate concentrations in geothermal channels are often higher at intermediate pH values (5.5–7) due to greater stability compared to low pH, where thiosulfate disproportionation to elemental sulfur and sulfite $\left({\text{SO}}_{\text{3}}^{\text{2}-}\right)$ can occur rapidly over time scales of seconds-minutes (Xu et al., 1998; Nordstrom et al., 2005). The impact of higher thiosulfate on archaeal communities is not known, but may provide flexibility for energy conservation through oxidative or reductive processes (Amend and Shock, 2001). The morphology of elemental S in these sulfidic sediments is generally rhombohedral, but spheres of variable diameter are also found, and cells have been observed adhering to these S minerals (e.g., Inskeep et al., 2010; Macur et al., 2013). The role of biota in the mineralization of FeS_2_ and Sb_2_S_3_ in JCHS_4 is not known, but does not require a reductive step since reduced constituents necessary for the formation of these minerals are already present in the source waters [i.e., Fe(II), DS; Table 1].

To contrast geochemical systems heavily influenced by sulfide and elemental S, two microbial mat samples were obtained from an acidic spring (pH 3.3–3.5) in the *One Hundred Spring Plain* (OSP), and included both a filamentous “streamer” community (OSP_14) and an Fe(III)-oxide microbial community (OSP_8) from the same geochemical environment. The “streamer” communities are infrequently distributed on top of the Fe-oxide mats from 70 to 80°C in shallow (∼1 cm), high-velocity outflow channels (Figure 1) (Takacs-Vesbach et al., 2013). The Fe-oxide mats (OSP_8) form as a result of oxidation of dissolved Fe(II) and subsequent deposition of amorphous, high-arsenate, Fe(III)-oxides (Inskeep et al., 2004; Macur et al., 2004). Prior metagenome and mRNA analysis of Fe(III)-oxide samples from *Beowulf Spring* confirmed the importance of *Metallosphaera*-like organisms and Fe(II)-oxidizing genes within these systems (Inskeep et al., 2010; Kozubal et al., 2011, 2012). The high-temperature Fe(III)-oxide mineralizing environments contain higher oxygen contents and support significantly greater archaeal diversity than low-pH (i.e., pH ∼ 2–6) sulfidic systems (Inskeep et al., 2005, 2010; Kozubal et al., 2012).

### Phylogenetic analysis of metagenome sequence

**Figure 2:**
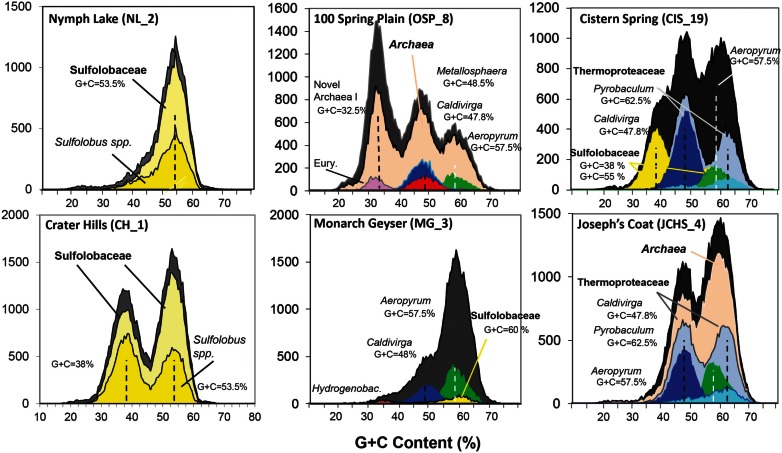
**Frequency plot of the G + C content (%) of random shotgun sequence reads (Sanger) obtained from archaeal habitats in Yellowstone National Park (YNP)**. Phylogenetic classification of each sequence (∼800 bp) was performed using MEGAN (“blastx”), which shows the predominant populations that contribute to metagenome sequence in these environments (dark-gray = total reads, pink = domain *Archaea* (shown in sites OSP_8 and JCHS_4 only), yellow = *Sulfolobaceae* (identity to *Sulfolobus* sp. also shown), red = *Metallosphaera* sp., green = *Aeropyrum pernix*, violet = Euryarchaeota, light-gray = Thermoproteaceae, dark-blue = *Caldivirga* sp., light-blue = *Pyrobaculum* sp.). Site WS_18 is shown in Figure A1 in Appendix.

Analysis of individual sequences (average length ∼800 bp) across these chemotrophic environments revealed systems inhabited by as few as one dominant population type (e.g., NL_2) to those containing significant archaeal diversity (e.g., OSP_8, WS_18). Combined phylogenetic (MEGAN) and G + C content (%) analysis of all individual sequences revealed the predominant phylotypes represented in each site (Figure 2). Sequences from the acidic and sulfidic sites (CH_1 and NL_2) were dominated by members of the order Sulfolobales (family *Sulfolobaceae*). The single dominant population type in NL_2 with a G + C content of ∼53.5% (referred to here as Type 1 Sulfolobales) was also one of two main population types present in CH_1 (Figure 2). A second *Sulfolobaceae* population in CH_1 (Type 2 Sulfolobales, G + C = 38%) was also found in the sulfur sediments at *Cistern Spring* (CIS_19) (along with less-dominant Type 1 populations). Conversely, *Monarch Geyser* (MG_3) contained a smaller number of Sulfolobales-like sequence reads with G + C contents near 60% (Figure 2). Sequence reads in CH_1 and NL_2 were classified at two different levels of phylogenetic resolution (family and genus) to illustrate that the total archaeal reads (gray) were nearly all related to the family *Sulfolobaceae*, but that considerably fewer sequences were highly related to the reference genomes of *Sulfolobus* spp. (Figure 2).

**Figure A1:**
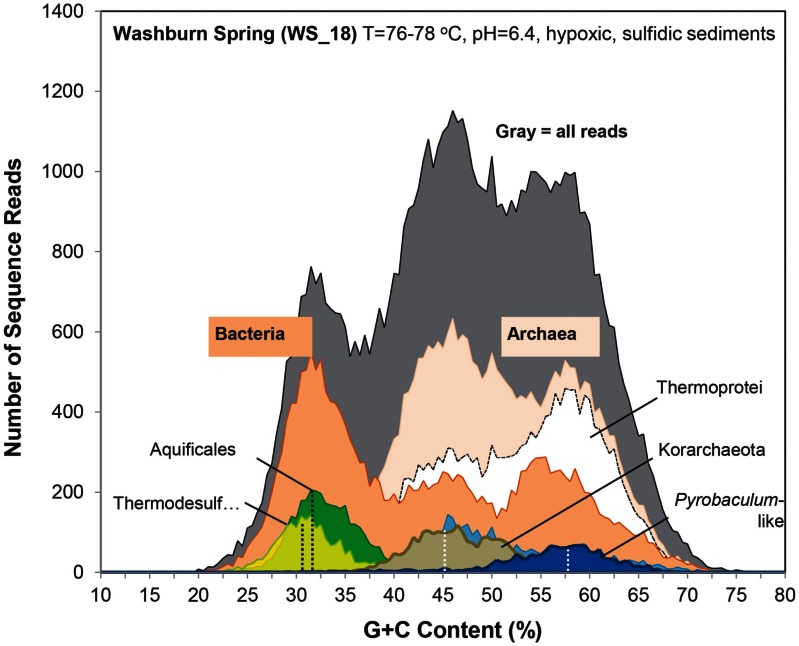
**G + C content (%) frequency plot of random shotgun sequence reads (Sanger) obtained from Washburn Springs (WS_18) sediments rich in elemental sulfur and pyrite**. Phylogenetic classification of each sequence read (∼800 bp) was performed using MEGAN (“blastx” analysis) (gray = total reads; orange = domain *Bacteria*; light-pink = domain *Archaea*; dark-green = Aquificales (primarily *Sulfurihydrogenibium*-like); light-green/yellow = class Thermodesulfobacteria; bronze = phylum Korarchaeota; white = class Thermoprotei; dark-blue = *Pyrobaculum*-like; light-blue = *Thermofilum*-like; other minor assignments with <100 total reads are not shown).

Metagenome sequences from the less acidic, sulfidic sediments of *Monarch Geyser* (MG_3), *Cistern Spring* (CIS_19), and *Joseph’s Coat Springs* (JCHS_4) were dominated by populations within the orders Thermoproteales and Desulfurococcales (phylum Crenarchaeota). The G + C content (%) of the predominant Desulfurococcales population was consistently ∼59% across all sites (Figure 2), while the two Thermoproteales populations exhibited G + C contents of either ∼48% (Type 1, light-blue; *Caldivirga/Vulcanisaeta*; e.g., Itoh et al., 1999, 2002), or ∼62.5% (Type 2, dark-blue; *Pyrobaculum* clade; e.g., Volkl et al., 1993; Fitz-Gibbon et al., 2002). The sulfidic sediments at WS_18 (pH 6.4) also contained representatives of the Thermoproteales (e.g., a Type 2 *Pyrobaculum-*like population, and a Type 3 *Thermofilum*-like population), as well as significant fractions of *Sulfurihydrogenibium* spp. (Aquificales), Thermodesulfobacteria, and members of the Korarchaeota (G + C ∼ 40–50%) (Figure A1 in Appendix).

The acidic Fe(III)-oxide microbial mat from *One Hundred Spring Plain* (OSP_8) contained considerable archaeal diversity, which correlated with low sulfide (Table 1) and higher levels of dissolved oxygen [i.e., 30–40 μM O_2_(aq)]. At least five distinct populations are evident in the G + C (%) distribution plot (Figure 2); phylogenetic analysis showed that these peaks corresponded to phylum Euryarchaeota (G + C ∼ 31%), *Vulcanisaeta/Caldivirga* sp. (G + C ∼ 47.8%), *Metallosphaera* sp. (G + C ∼ 48.2%), *Acidilobus* sp. (G + C ∼ 57.5%), and a novel archaeal population with a G + C content of 32.5 ± 2%. The sequences of the novel archaeal Group I population have been proposed (Kozubal et al., 2013) to represent a new phylum-level lineage in the *Archaea*, the Geoarchaeota. The sequence reads similar to *Aeropyrum*-like populations are actually more closely related to the recently released genome of *Acidilobus saccharovorans* (Prokofeva et al., 2009; Mardanov et al., 2010) and draft genome sequence for *A. sulfurireducens* (Boyd et al., 2007; Inskeep et al., 2010), but the *Aeropyrum* reference correctly identifies this population as a member of the order Desulfurococcales.

### Phylogenetic analysis of metagenome sequence assemblies

**Figure 3:**
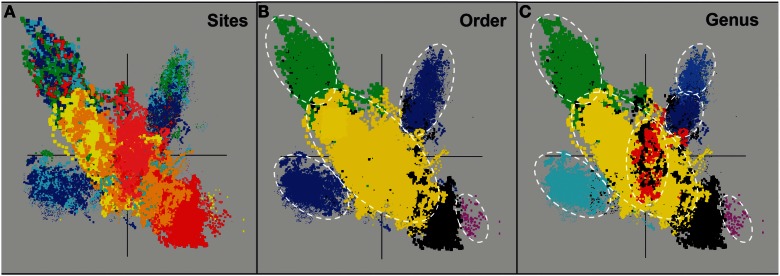
**Nucleotide word frequency PCA plots of metagenome assemblies (>5 kb contigs) from high-temperature, archaeal-dominated geothermal environments in YNP**. **(A)** Sites: *Crater Hills* (CH_1) = gold; *Nymph Lake* (NL_2) = yellow; *Monarch Geyser* (MG_3) = green; *Joseph’s Coat Hot Springs* (JCHS_4) = dark-blue; *Cistern Spring* (CIS_19) = light-blue; *One Hundred Spring Plain* “streamer” (OSP_14) = light-red; *One Hundred Spring Plain* Fe-oxide mat (OSP_8) = red; **(B)** Order Level: The identical PCA orientation was maintained as presented in A, but with phylogenetic analysis of contigs to the closest reference genome (yellow = Sulfolobales; green = Desulfurococcales; blue = Thermoproteales; purple = Nanoarchaeum; black = unassigned); and **(C)** Genus Level (yellow = *Sulfolobus*; red = *Metallosphaera*; green = *Aeropyrum*; light-blue = *Pyrobaculum*; dark-blue = *Vulcanisaeta*; gray-blue = *Caldivirga*; purple = *Nanoarchaeum*; black = unassigned).

Assembly of individual sequence reads resulted in large contigs and scaffolds for several of the predominant archaeal populations present in these sites. Sequence assemblies for each sample were evaluated using nucleotide word frequencies (NWF) (Teeling et al., 2004) combined with Principal Components Analysis (PCA; Figure 3). This technique can often resolve organisms at the genus-species level because of unique sequence character including G + C content (%) and codon usage bias (Teeling et al., 2004; Inskeep et al., 2010). Assemblies from archaeal sites were separable to a large extent using principal component analysis (Figure 3A). The PCA plot is also shown with corresponding phylogenetic analysis of contigs Automated Phylogenetic Inference System (APIS; Badger et al., 2006) at the order-level (Figure 3B), and the genus-level (Figure 3C). The majority of predominant phylotypes present across these communities were delineated using this approach.

**Figure 4:**
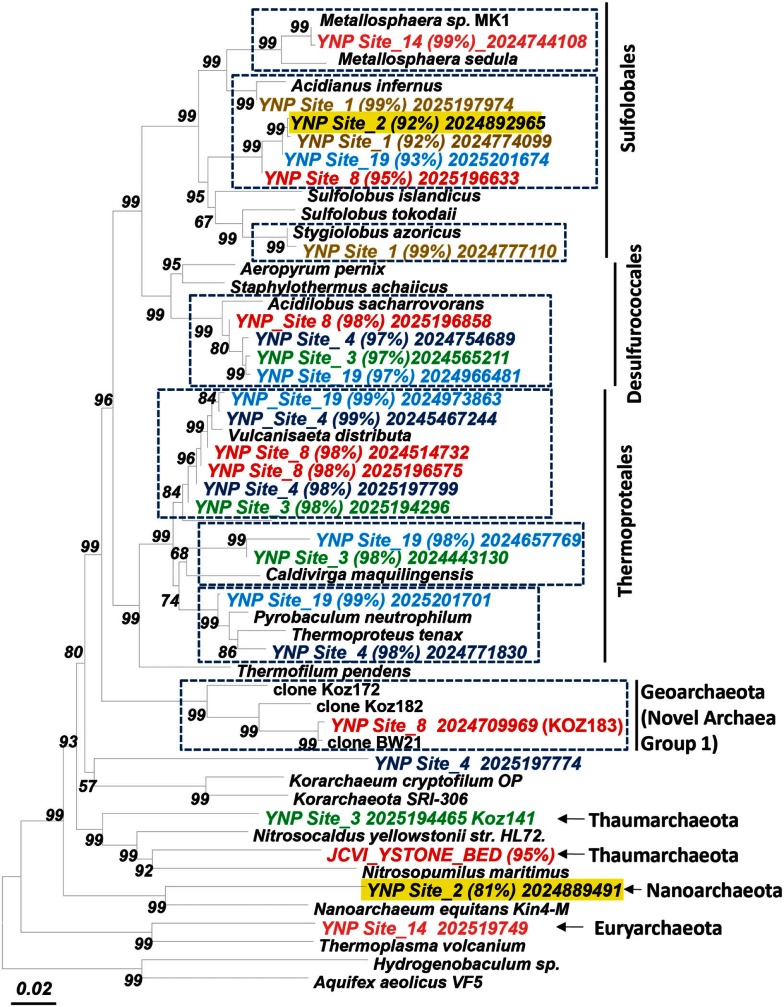
**Phylogenetic tree (16S rRNA gene) showing major phylotypes identified in assembled metagenome sequence across high-temperature sites dominated by archaea**. Entries from metagenome sequence are labeled and colored by site (% nucleotide identity to closest cultivated relative; scaffold ID; dashed boxes indicate the dominant sequence types characterized in this study; bootstrap values based on neighbor joining tree with 1,000 replications).

The predominant sequence assemblies corresponded to the major peaks observed in the G + C distribution plots (Figure 2). For example, the acidic sites were dominated by Sulfolobales populations, and three major lineages within this order were identified across sites (Table S2 in Supplementary Material). The acidic elemental sulfur-rich sediments (CH_1 and NL_2) contained one and two major Sulfolobales types, respectively. These populations are related to *Sulfolobus* spp. (Type 1, G + C content = 52–54%) and *Stygiolobus* spp. (Type 2, G + C content = 38%) based on full-length genes identified in the assembled sequence (including the 16S rRNA gene) (Figure 4; Tables S1 and S2 in Supplemental Material). The higher pH (pH 4–6) sulfur sediments from MG_3, CIS_19, and JCHS_4 also contain variable amounts of sequence corresponding to Sulfolobales populations (Figures 3A,B); the G + C peak in CIS_19 at 36–38% was similar to the *Stygiolobus* population in CH_1 (Figure 2). Conversely, the Fe-oxide mats (OSP_8) and Fe-oxide “streamer” community (OSP_14) were the only sites to contain sequence data corresponding to *M. yellowstonensis*-like populations (Figure 3C). This aerobic organism has been shown to oxidize Fe(II) using a different terminal oxidase complex (*fox*) than used for S oxidation (*dox*) (Bathe and Norris, 2007) and can generate sufficient energy for growth by oxidizing large amounts of Fe(II) (Kozubal et al., 2011).

The main sequence clusters observed in MG_3, CIS_19, and JCHS_4 were related to members of the orders Desulfurococcales and Thermoproteales (Figure 3). Phylogenetic analysis consistently showed two major types of Thermoproteales in both CIS_19 and JCHS_4 (and to a lesser extent in MG_3 and OSP_8) (Figure 3B), corresponding to *Caldivirga/Vulcanisaeta*-like (Type 1 Thermoproteales) and *Pyrobaculum*-like (Type 2 Thermoproteales) organisms (Figure 3C). Similar *Caldivirga/Vulcanisaeta* and similar *Acidilobus-*like populations were also observed in the non-sulfidic, Fe-oxide mat (OSP_8) (based on NWF PCA, sequence similarity, G + C content, and functional analysis). Consequently, although these populations were clearly the main community members detected in hypoxic sulfur-rich sediments from pH 4 to 6, (Jay et al., 2011) they also appeared in Fe-oxide mats where sulfide and elemental sulfur are generally below detection.

At least four major archaeal populations were identified in the Fe-oxide mat (OSP_8) using G + C content and NWF PCA analysis: *M. yellowstonensis*, *Vulcanisaeta* spp., *Acidilobus* spp. and a “novel archaeal Group I” population belonging to the proposed phylum, Geoarchaeota (Kozubal et al., 2013). The deeply rooted phylogenetic position of the 16S rRNA gene (Figure 4) was consistent with analysis of other single-copy genes (e.g., RNA polymerases, gyrases, transcriptional factors, etc.) identified within the assembled sequence (Table S4 in Supplementary Material). The metagenome sequence of NAG1 was only distantly related to other reference genomes; amino acid identities relative to currently available reference genomes generally ranged from 40 to 60%, and closest relatives of individual genes included members of the domain *Archaea* as well as *Bacteria* (Kozubal et al., 2013). The Geoarchaeota (NAG1) population was the most abundant community member in OSP_8, which resulted in excellent contig coverage (average ∼6×), and a total scaffold length of ∼1.7 Mb in only eight scaffolds (Kozubal et al., 2013). The Fe-oxide community (OSP_8) also contained several other archaea (although at lower coverage) including relatives of the Euryarchaeota (distantly related to the Thermoplasmatales), Nanoarchaeota, Crenarchaeota (i.e., other Sulfolobales), as well as the Candidate phylum Thaumarchaeota (Brochier-Armanet et al., 2008; Beam et al., 2011).

#### Nanoarchaeal sequence

Assembled sequence distantly related to *Nanoarchaeum equitans* (Huber et al., 2002; Waters et al., 2003) was found in several archaeal-dominated microbial communities (Figure 4; Table S1 in Supplemental Material). Partial 16S rRNA gene sequences (among other single-copy genes; Table S4 in Supplemental Material) corresponding to the Nanoarchaeota were observed in assembled sequence from sulfidic sediments (NL_2, JCHS_4) and Fe-oxide mats (OSP_8), although these are only distantly related to *N. equitans* (∼82–84% similarity, Table S1 in Supplemental Material). Given that *Ignicoccus hospitalis* is not an important member of these archaeal communities, either other hosts are important to these nanoarchaea, or they may be free-living. Insufficient coverage of these novel nanoarchaea does not allow a thorough genomic evaluation; however, nearly 100 kb of assembled sequence was obtained for nanoarchaea present in the Fe-oxide mat samples (OSP_8 and 14). The average G + C content of the nanoarchaeal sequence is ∼27%, considerably lower than observed for *N. equitans* (31.6%). Further work will be necessary to fully appreciate the diversity of nanoarchaea in thermal systems of YNP and determine whether the extensive distribution of different nanoarchaeal sequences (Hohn et al., 2002; Casanueva et al., 2008; Clingenpeel et al., 2011) implies a corresponding diversity of host species.

#### Assembly of viral genomes

**Figure 5:**
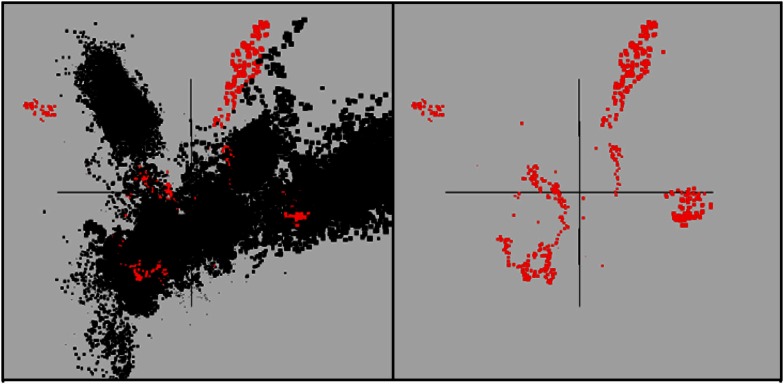
**Nucleotide word frequency PCA scatter plots of non-viral (black) versus viral (red) scaffolds identified within the metagenome assemblies of archaeal-dominated sites (Table 2 provides additional details on the characteristics of viral scaffolds)**.

In total, 10 scaffolds from the archaeal-dominated samples were classified as “viral,” based on phylogenetic analysis of known viruses (Table 2; Figure 5). Although the similarity of these scaffolds to known viruses varied considerably, the Thermoproteus spherical-like viruses found in sites NL_2, JCHS_4, and CIS_19 are highly similar to and nearly the same length as known isolates. Others such as scf_6649105 and scf_5653402 had very weak matches to predicted viral proteins. The viruses found in these samples are related (if only distantly) to other archaeal viruses and thus consistent with expectation. Two sets of viral scaffolds (Group A and B) were found in more than one sample (Table 2), and in both cases the sequences were highly similar (92%+ nucleotide identity). CRISPR regions including both spacer regions and direct repeats (DR) (Grissa et al., 2007; Makarova et al., 2011) were predicted from these assemblies as well as assemblies generated from the same habitat types sampled ∼1 year prior to the current study (Inskeep et al., 2010). Near perfect alignments were found between CRISPR spacer regions and 8 of the 10 viral-like scaffolds (Table 2). CRISPR spacer regions from the prior project matched two of the scaffolds identified here implying some continuity between the viral and microbial populations. A total of 5,435 CRISPR spacers were identified from the archaeal samples, and only 16 of these matched the scaffolds annotated as viral (one mismatch allowed). Even relaxing the alignment parameters (three mismatches allowed) only increased this number modestly to 26. A total of 382 spacers have matches to sequences not annotated as viral. Those with multiple matches were examined, but could not be verified as viral due to their short length. Consequently, although the majority of spacer regions identified within CRISPR elements were not recognized as viral, this may be due to our inability to recognize novel and potentially dynamic viral sequence.

**Table 2:** **Scaffolds with similarity to known viruses**.

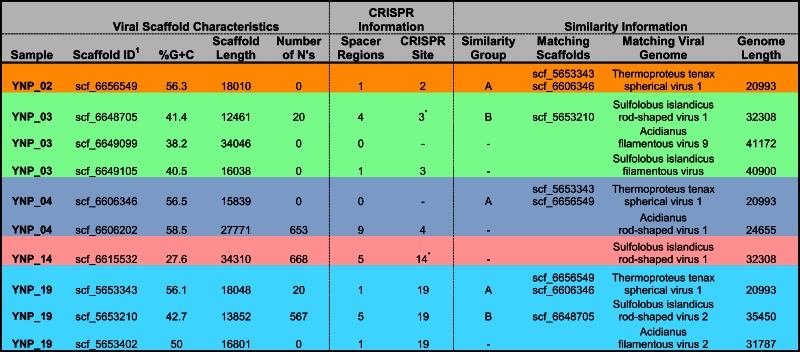

*^1^Complete scaffold ID (_ = 111868) retained as an identifier in sequence assemblies deposited with IMG/M*.

*The source of the assembled reads, %G + C, scaffold length, and the number of ambiguous bases (N’s) in “viral-related” scaffolds are shown. Scaffolds showing nucleotide sequence similarity to each other are identified by the “Similarity Groups” (A, B), where the similar scaffolds are listed. The best match to known viral genomes (BLASTX to the NRAA database) is shown along with the length of that genome. Sequence from eight of these scaffolds contained matches to CRISPR spacer regions (three mismatches allowed)*.

We found similar patterns in the diversity and distribution of CRISPR DR across the archaeal-dominated sites (Table S3 in Supplementary Material) corresponding to the dominant phylotypes present (e.g., Figure 4). For example, DR sequences classified as most similar to reference Sulfolobales sequence were found distributed across the archaeal sites containing these organisms (CH_1, NL_2, MG_3, OSP_8, and CIS_19). Moreover, sites dominated by a particular phylotype such as the Sulfolobales (e.g., CH_1, NL_2) only contained Sulfolobus-like DR sequences. Sites containing greater crenarchaeal diversity such as CIS_19 and JCHS_4 reveal a significant number of DRs contributed from *Sulfolobus*, *Caldivirga*, and *Pyrobaculum*-like populations, consistent with the dominant populations identified using standard phylogenetic markers. DRs were not found in any of the four replicate Desulfurococcales populations observed from pH 3 to 6 (sites OSP_8, MG_3, JCHS_4, CIS_19). The different “*Thermofilum*-like” DR sequences observed in OSP_8 and WS_18 (Table S3 in Supplementary Material) are each unique to the study; those from site OSP_8 are contributed from the NAG1 population (Candidate phylum Geoarchaeota; Kozubal et al., 2013).

#### Phylogenetic summary

Manually curated scaffolds/contigs corresponding to seven major archaeal phylotypes (or ∼15 *de novo* assemblies including replicates) were obtained from the metagenome sequence (Table S2 in Supplementary Material). Replicate *de novo* assemblies of similar phylotypes were obtained for *Metallosphaera*-like populations (OSP_8 and 14), *Caldivirga/Vulcanisaeta* types (MG_3, CIS_19, JCHS_4, and OSP_8), *Acidilobus*-like organisms (MG_3, CIS_19, JCHS_4, and OSP_8), *Sulfolobus and Stygiolobus*-like populations (CH_1, NL_2, and CIS_19), and *Pyrobaculum* types (CIS_19, JCHS_4, and WS_18). The amount of assembled sequence obtained for many of these indigenous archaeal populations was greater than 1 Mbp (Table S2 in Supplementary Material), and may represent close to expected genome sizes based on sequenced archaeal relatives. Each contig within each sequence cluster (NWF analysis) was carefully screened using G + C content (%) combined with BLAST scores and functional relevance. Fragment recruitment plots, coverage estimates and evaluation of single-copy genes suggests that the assembled sequence represents near complete (>90%) genomic sequence for several (6–8) of these phylotypes. A modest survey of single-copy genes corresponding to the predominant populations present in these archaeal sites provides a summary of the possible completeness represented in the assembled sequence (Table S4 in Supplementary Material) and also provides insight regarding the possible variation existing within closely related populations. Given that our analysis was limited to near-full length genes, additional single-copy genes corresponding to these populations may be present in small contigs or individual sequences that did not assemble well, and these may also be extremely useful for understanding more variable regions among individuals comprising these phylotypes.

### Protein family analysis of archaeal communities

**Figure 6:**
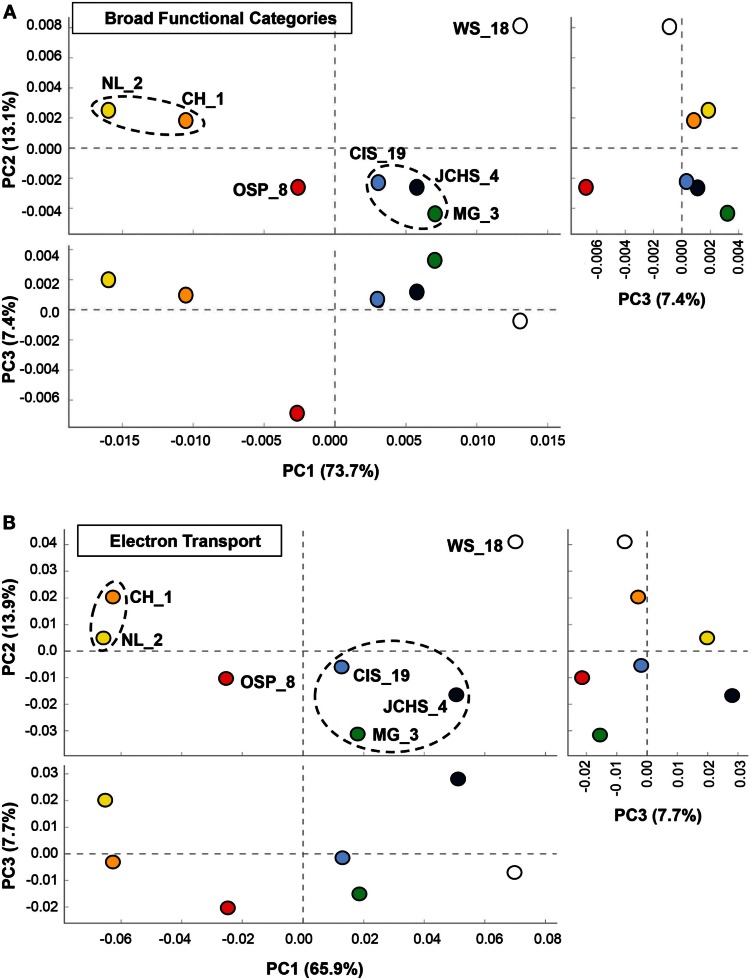
**Principal components analysis (PCA) of relative gene abundances across seven high-temperature archaeal-dominated chemotrophic communities**. The three panels show pairwise plots of the first three principal components (PC1 and PC2 account for 76% of the variation across sites, while PC3 only represents ∼6%). **(A)** All TIGRFAMs grouped into functional categories, and **(B)**. Only those TIGRFAMs associated with the role category “Electron Transport.” Sites are colored as before: *Crater Hills* (CH_1) = gold; *Nymph Lake* (NL_2) = yellow; *Monarch Geyser* (MG_3) = green; *Joseph’s Coat Hot Springs* (JCHS_4) = dark-blue; *Cistern Spring* (CIS_19) = light-blue; *One Hundred Spring Plain* (OSP_8) = red; *Washburn Spring* (WS_18) = open.

One of the primary aims of the study was to identify specific metabolic attributes of individual archaeal populations found distributed across chemotrophic habitats, and determine if functional attributes of these communities correlated with specific geochemical properties. Moreover, a thorough evaluation of metabolic capability provides a direct understanding of which oxidation-reduction reactions may be driving productivity in these chemotrophic habitats, and how the functional capabilities of different and/or very similar organisms may vary in response to specific environmental parameters. The abundances of all proteins identified in the assembled metagenome sequence data were evaluated using PCA and hierarchical clustering to compare relative differences and/or similarities among sites. PCA of relative gene abundances across all TIGRFAMS grouped into functional categories showed strong similarity between individual sites with similar phyla (Figure 6A). Factor 1 (accounting for ∼74% of the relative TIGRFAM variation across sites) separated sites based roughly on the relative abundance of Sulfolobales (which also tracks with site pH), where CH_1 and NL_2 were dominated by only two population types of Sulfolobales and WS_18 contained little to no Sulfolobales. Sites that contained a greater abundance of Desulfurococcales and Thermoproteales (mildly acidic sulfur sediments, MG_3, CIS_19, JCHS_4; Macur et al., 2013) also clustered together. Site OSP_8 (oxic, no sulfide) contained multiple archaeal populations (including the new Geoarchaeota) and plotted separate from all other sites. Principal components factors 2 and 3 were less important in describing functional variation across the archaeal sites (only 13 and 7%, respectively); however, PC3 results in separation of OSP_8 relative to all other archaeal sites. OSP_8 was the only oxic habitat included in this study and was the only site that contained the NAG1 population (Candidate phylum Geoarchaeota, Kozubal et al., 2013).

**Figure 7:**
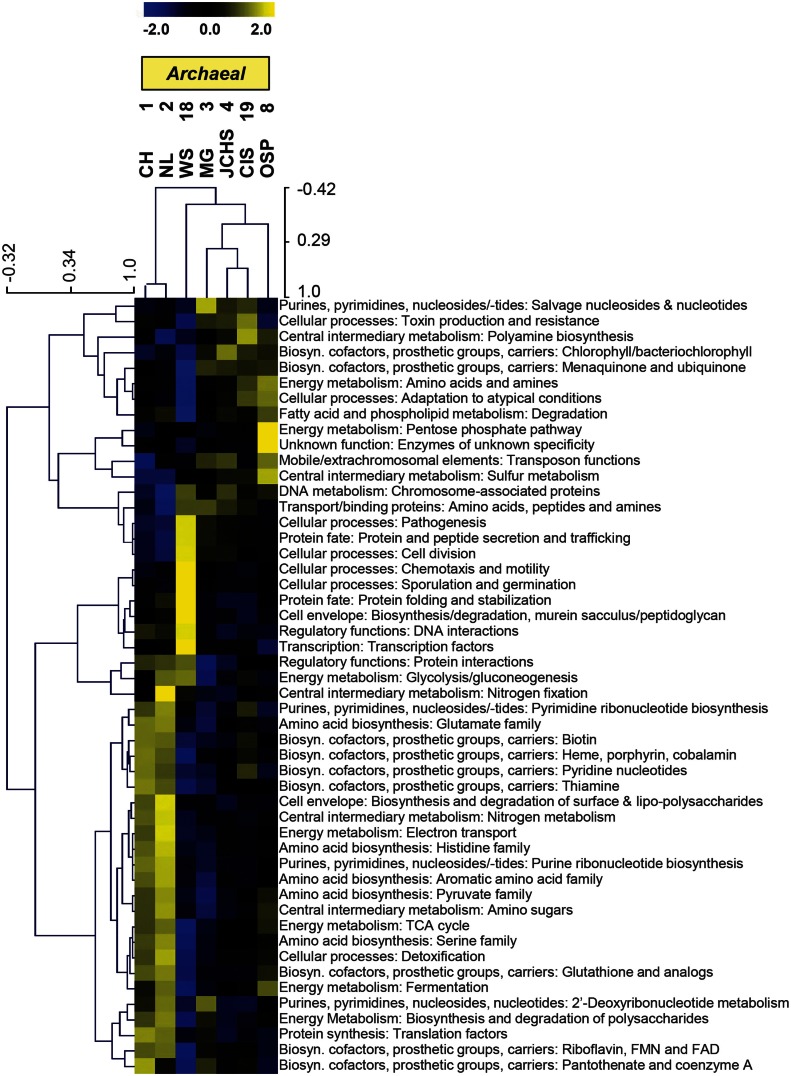
**Hierarchical cluster analysis of relative gene abundances across seven archaeal-dominated communities using all TIGRFAMs grouped into functional categories**. Broad TIGRFAM categories include all cellular processes such as regulatory functions, energy metabolism, central C metabolism, mobile elements, transcription, cofactors, and transporters. Data was standardized by functional category before clustering to avoid biasing analysis by a few categories with high gene abundance. Pearson correlation was used as the distance measure for average linkage agglomerative clustering. TIGRFAMs from WS_18 and OSP_8 form separate functional clades consistent with the phylogenetic uniqueness of these sites.

The TIGRFAM categories responsible for observed differences across these archaeal sites were evaluated in more detail using hierarchical clustering (Figure 7). The site clustering is consistent with PCA separation (Figure 6A), and correlates with environmental factors including pH and dissolved sulfide/oxygen. Examples of TIGRFAM categories most different across sites include processes related to sulfur metabolism, peptide secretion, electron transport, fermentation, biosynthesis of cofactors, and routine cellular processes including cell division, sporulation, and motility (Figure 7). Given the substantial phylogenetic differences across sites, it is not surprising that the relative differences within and across TIGRFAM categories retained these signatures. However, it can be difficult to appreciate specific functional differences using broad TIGRFAM categories, and each protein identified in the metagenome sequence must be studied independently to verify putative functional assignment and understand aspects related to gene neighborhood and pathway context.

**Figure A2:**
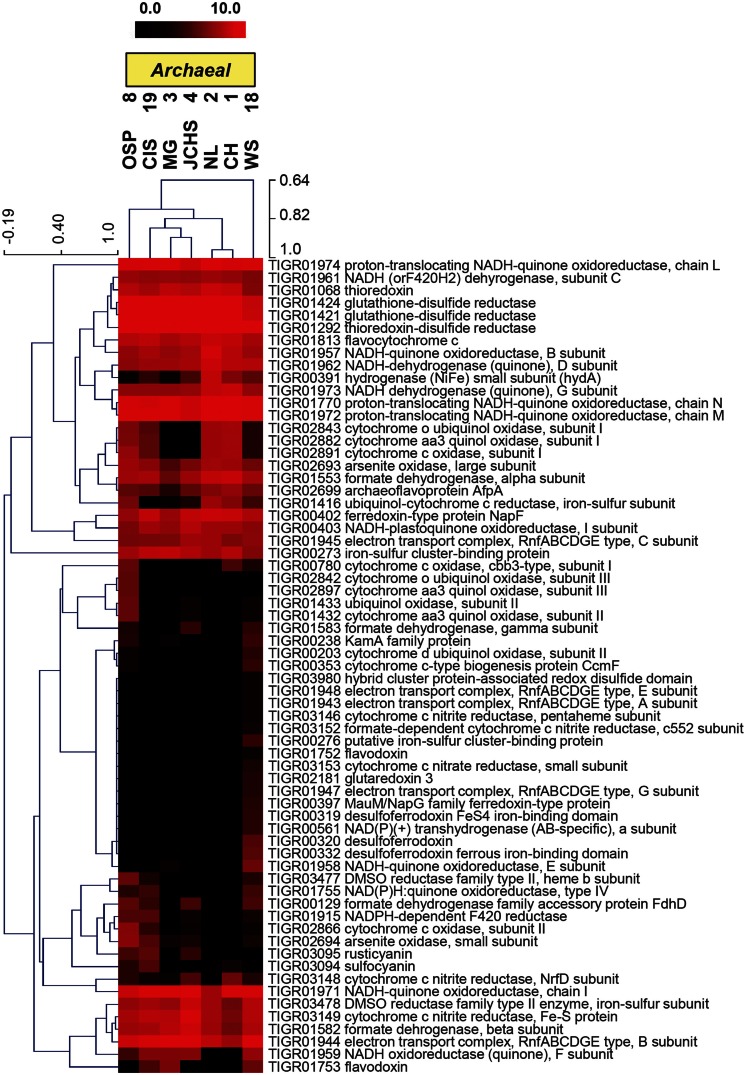
**Hierarchical cluster analysis of relative gene abundances in the TIGRFAM role category “Electron Transport” across seven archaeal-dominated geothermal communities**. TIGRFAM gene families with low variation across the sites were removed before the clustering to retain ∼50 of the most variable families. Subunits of the protein complexes were only represented by one representative TIGRFAM family. Pearson correlation was used as the distance measure for average linkage agglomerative clustering.

More detailed comparisons among sites were made using protein hits confined to specific TIGRFAM categories. For example, the relative abundances of TIGRFAM assignments within the category “Electron Transport” resulted in consistent site separation as observed using broad categories (Figure 6B), however, the specific TIGRFAMs in this category provide greater insights into microbial processes that are influenced directly by geochemical conditions such as the presence of sulfide versus oxygen. Indeed, numerous “Electron Transport” TIGRFAMs with the greatest relative differences across sites relate to the types of cytochromes, oxygen reductases, sulfur reductases, hydrogenases, or other respiratory proteins present in the metagenome sequence. For example, the ubiquity of heme Cu oxidases (Type 1 HCO; García-Horsman et al., 1994; Kozubal et al., 2011) in *M. yellowstonensis* [an aerobic Fe(II)-oxidizing Sulfolobales] and the NAG1 population (Candidate phylum Geoarchaeota) present in OSP_8, was in contrast to the notable absence of these respiratory proteins in the Desulfurococcales and Thermoproteales populations that dominate hypoxic, mildly acidic sulfur sediments (Figure A2 in Appendix). Comparison of “Electron Transport” TIGRFAMs emphasized differences in OSP_8 versus WS_18 compared to all other sites, due in large part to the oxic nature of Fe(III)-oxide microbial mats, and to the considerably higher abundance of bacterial pathways in WS_18 (Thermodesulfobacteria, *Sulfurihydrogenibium*, and higher G + C bacteria represent a significant proportion of the total sequences from WS_18; Figure A1 in Appendix). Respiratory processes in these bacteria are considerably different than the dominant archaea present in other sites, which contained very few bacterial reads (less than 10% on average). Consequently, although comparison of relative TIGRFAM abundances across sites represents functional differences of individual phylotypes, a detailed functional analysis of these populations provides clarification regarding observed functional differences across sites.

### Functional analysis of predominant archaeal phylotypes

The archaeal populations detected within these high-temperature sites exhibit extensive differences in energy conservation and CO_2_ fixation pathways. To obtain more detailed information on specific functional genes present across sites, an extensive list of query genes coding for putative proteins important in CO_2_ fixation pathways, electron transport and trace-element detoxification (As, Hg, superoxide) was compared to the assembled metagenomes (Table 3). All positive sequence hits were then compared to reference databases (using “blastp”), and analyzed individually (e.g., homology scores, phylogenetic trees) prior to confident assignment (Table 3). The detailed inventory of specific functional genes is consistent with TIGRFAM protein family assignments, but is focused on specific pathways/proteins associated with geochemical processes.

**Table 3:** **Summary of key metabolic genes identified in sequence assemblies of the predominant archaeal populations present across sites, which exhibited a wide range in pH, dissolved sulfide, and dissolved oxygen (see Table 1)**.

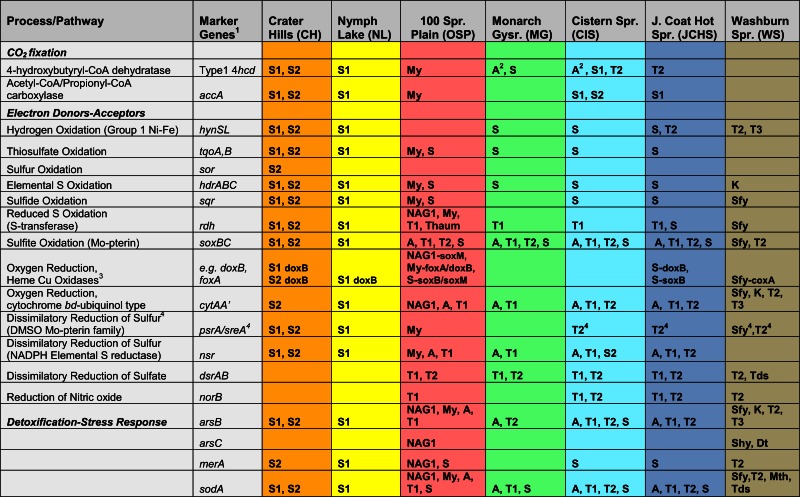

*Population types: S1 and S2, Sulfolobales Types 1 and 2, respectively; My, *M. yellowstonensis*; A, Desulfurococcales (*Acidilobus* sp.); T1, T2, T3, Thermoproteales. Types 1, 2, and 3, respectively; NAG1, “Novel Archaea Group I” (Candidate phylum Geoarchaeota; Kozubal et al., 2013); K, Korarchaeota; Sfy, *Sulfurihydrogenibium*-like; Dt, *Dictyoglomus*-like; Mth, Methanosarcinales; Tds, Thermodesulfobacteria*.

*^1^“High confidence” sequence matches to marker genes that code for proteins with high specificity for possible pathway. Query sequences used to search for specific functional genes given in Table S3 in Supplementary Material (Inskeep et al., 2013). Genes not present in these sites = amoA, nifH, mcrA, nirK, nirS, nosZ*.

*^2^Specific heme Cu oxidases (subunit 1) are listed for each population type*.

*^3^Uncharacterized DMSO-Mo-pterins belonging to the Thermoproteales (T2) and *Sulfurihydrogenibium* (Sfy) populations are listed here*.

#### Oxidation-reduction

The primary electron donors and acceptors that support metabolism of these archaeal phylotypes establish a critical link to geochemical processes. The oxidation of H_2_ is highly exergonic, and this is no exception in thermal habitats where concentrations of dissolved H_2_(aq) are considerable (Table 1) and represent an available energy source (Amend and Shock, 2001; Inskeep et al., 2005; Spear et al., 2005). However, evidence for Group I Ni–Fe hydrogenases (responsible for H_2_ uptake and oxidation to H^+^, Viginais and Billoud, 2007) across these sites is limited to Sulfolobales populations, with the exception of one full-length (large and small subunit) Ni–Fe hydrogenase (on same contig) belonging to one of the Thermoproteales populations (*Pyrobaculum*-like) from JCHS_4 (Table 3). Complete Ni–Fe hydrogenases were present in the Sulfolobales populations at CH_1 and NL_2, but were absent in the *M. yellowstonensis*-like population present in OSP_8 (or OSP_14), as well as all other predominant archaeal populations present in these sites. Moreover, no evidence was found for key marker genes associated with methanogenesis (*mcrA*) (Ferry, 1999; Dhillon et al., 2005) or the oxidation of methane (*pmoA*), arsenite (*aro*A/*asoA*/*aioA*) (Hamamura et al., 2009), or ammonium (*amoA*), despite the fact that (i) substrates for these enzymes were present in high concentrations, (ii) oxidation reactions with these potential donors are highly exergonic in these thermal environments (Amend and Shock, 2001; Inskeep et al., 2005), and (iii) other cultivated thaumarchaea have been shown to oxidize ammonia (e.g., Walker et al., 2010).

**Figure A3:**
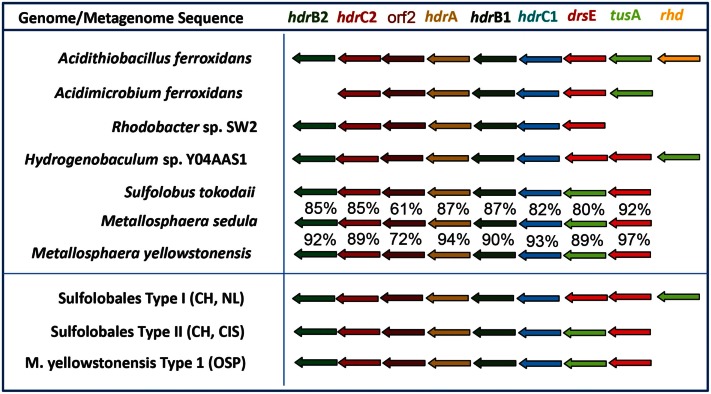
**Conservation of open reading frames found in the heterodisulfide reductase (HDR) gene complex of several acidophilic bacteria and archaea known to be utilized during the oxidation of reduced sulfur**. Highly syntenous components of the HDR-gene complex are conserved in several of the thermophilic archaea found in YNP, as well as bacteria within the order Aquificales (Takacs-Vesbach et al., 2013). Percent amino acid similarities of the deduced HDR proteins are shown for *M. sedula* and *M. yellowstonensis* relative to *S. tokodaii*. The Sulfolobales Type I and *Hydrogenobaculum* sp. Y04AAS1 have two copies of *drs*E [gene names: *hdr*B1/*hdr*B2 = heterodisulfide reductase, subunit B (COG2048); *hdr*C1/*hdr*C2 = heterodisulfide reductase, subunit C (COG1150); *orf*2 = hypothetical conserved protein; *hdr*A = heterodisulfide reductase, subunit A (COG1148); *dsr*E = peroxiredoxin family protein (COG2210); *tus*A = *sir*A family of regulatory proteins; *rhd* = rhodanese-related sulfurtransferase (COG0607)].

The oxidation of reduced sulfur species (i.e., sulfide, S, thiosulfate, sulfite) is also highly exergonic (Amend and Shock, 2001; Ghosh and Dam, 2009). Genes (or gene complexes) known to code for proteins that catalyze the oxidation of reduced forms of S were found in several of the predominant archaeal populations present in these chemotrophic sites, especially members of the Sulfolobales (Table 3). All of the Sulfolobales populations (Types 1, 2, and *M. yellowstonensis*-like) contained gene complexes that are highly syntenous to and homologous to the heterodisulfide reductase (HDR) gene complex (Figure A3 in Appendix) found in other Sulfolobales and bacterial genomes (Auernik and Kelly, 2008; Quatrini et al., 2009). The HDR complex is comprised of several heterodisulfide proteins and accessory components, which have been proposed to oxidize elemental sulfur to sulfite, followed by electron transfer to the quinol pool and ultimately to either a *bd*-ubiquinol oxygen reductase or a terminal oxidase complex (i.e., heme Cu oxidase). Genes coding for rhodanese domain proteins are also linked with the HDR complex, consistent with their putative role as sulfuryl-transferases (Hedderich et al., 2005; Quatrini et al., 2009). These populations also contain sulfide:quinone reductase genes (*sqr*) shown to code for proteins involved in the oxidation of H_2_S, HS^−^, and S^2−^ to S^0^ or polysulfide chains, followed by electron transfer to the quinone pool through a flavin adenine dinucleotide (FAD) cofactor (Cherney et al., 2010).

Moreover, genes known to code for proteins important in the oxidation of thiosulfate (TqoAB) were present in each of the major Sulfolobales types observed across these habitats (Table 3). A separate sulfur:oxygen reductase (*sor*) gene was also found in the Sulfolobales Type 2 population (*Stygiolobus*-like), and is the only archaeal population among these sites that appears to contain this gene, which has been implicated in the oxidation of elemental S in *Acidianus* spp. (Kletzin et al., 2004; Li et al., 2008). Nearly all archaea in these sites contained a sulfite oxidase molybdopterin, responsible for the oxidation of sulfite to sulfate (Table 3). However, the potential for oxidation of reduced S species (i.e., sulfide, elemental S, thiosulfate) was prevalent in the Sulfolobales populations, and these same genes were notably absent in members of the Desulfurococcales, Thermoproteales, and other archaeal populations present in these sites.

#### Respiratory pathways

The assembled metagenomes were also searched intensively for genes associated with aerobic and anaerobic respiration (Table 3). The presence of heme Cu oxidases (HCO; subunit I of terminal oxidase complexes) is an excellent indication of the potential to respire on O_2_ (García-Horsman et al., 1994). The majority of heme Cu oxidases were associated with the three Sulfolobales populations (S1, S2, and My; Table 3) as well as the Geoarchaeota population (NAG1) from OSP_8 (Kozubal et al., 2013). The Thermoproteales (T1, T2), Desulfurococcales (A), and Korarchaeota (K) present across these sites showed no evidence of HCO-type oxygen reductases; however, these assemblies contained either *cyt*AA′ or *cyt*AB type *bd*-ubiquinol oxidases (Table 3) that may represent high-affinity oxygen reductases important under hypoxic conditions (as observed in MG_3, CIS_19, JCHS_4, and WS_18; Table 1), or as O_2_ scavenging proteins (García-Horsman et al., 1994; Das et al., 2005).

The predominant Thermoproteales populations present in sites MG_3, CIS_19, JCHS_4, WS_18, and OSP_8 (i.e., Type I *Caldivirga/Vulcanisaeta* and Type 2 *Pyrobaculum/Thermoproteus*-like organisms) were the only archaea in this study to contain putative dissimilatory sulfite/sulfate reductases (DsrAB). Moreover, these populations contain the only *nor*B (nitric oxide reductase) genes found among these sites. No evidence of *nar*G, *nir*K, or *nir*S homologs were found, so it is unclear how these organisms might reduce nitrate to nitric oxide, which would be required prior to reduction of NO to N_2_O if using nitrate or nitrite as an electron acceptor (González et al., 2006). The NorB heme Cu oxidases could also play a role in reducing O_2_ (Flock et al., 2005), detoxifying NO (Watmough et al., 2009), or possible dismutation of NO (Ettwig et al., 2012).

Other possible electron acceptors used by these archaeal populations include elemental sulfur (S^0^) or polysulfides (Table 3). DMSO-molybdopterin sulfur reductase genes (*sre*A; Laska et al., 2003; Schut et al., 2007) were found in the Sulfolobales populations (S1, S2, My); however, the only other DMSO-molybdopterin genes found in these assemblies included formate dehydrogenases (*fdh*) and putative phenylacetyl CoA:acceptor oxidoreductases, which have been shown to be important in the oxidation of phenylacetic acids without using molecular oxygen (Rhee and Fuchs, 1999). These and several other novel DMSO-molybdopterin genes were consistently observed in the Thermoproteales T2 populations in MG_3, JCHS_4, CIS_19, and WS_18. Deduced proteins coded by these novel DMSO Mo-pterin genes do not cluster with currently known ArrA or SreA/PsrA proteins; consequently, it is not clear whether they play an important role in energy conservation for the Thermoproteales populations (Jay et al., 2011). The presence of a NAD(P)H elemental sulfur reductase (*nsr*) similar to that described in *Pyrococcus furiosus* (Blumentals et al., 1990; Schut et al., 2007) may represent an additional pathway for reduction of elemental sulfur (or polysulfides) in archaea, and copies of this gene were present in all of the major archaeal populations in these sites, with the exception of the Geoarchaeota (NAG1) population from OSP_8 (Table 3).

#### Carbon dioxide fixation

Evidence for CO_2_ fixation in the archaea has focused primarily on the recently discovered 3-hydroxypropionate/4-hydroxybutyrate (3HP–4HB) pathway, originally reported in *M. sedula* (Berg et al., 2007). The marker genes for this complex, 16-step pathway are (i) 4-hydroxybutyryl-CoA dehydratase (*4hbd*), which catalyzes the conversion of 4-hydroxybutyryl-CoA to crotonyl-CoA, and (ii) the bifunctional acetyl-CoA/propionyl-CoA carboxylase (AccB/AccC/PccB) (Hügler et al., 2003; Berg et al., 2007, 2010a,b). Excellent matches to these genes were found distributed throughout the Sulfolobales populations (Types S1, S2, and My; Table 3), and it is likely that these organisms are capable of fixing CO_2_. However, no *accA-*like genes were found in other archaea besides the Sulfolobales. Copies of a *4hbd* gene were observed in the *Acidilobus*-like (A) and *Pyrobaculum*-like (T2) populations in several sites (Table 3); however, despite the presence of other common metabolic genes in the “dicarboxylate” branch of the “dicarboxylate/4*hbd*” pathway, it is not clear from the metagenome assemblies that these organisms have the necessary genes for a complete CO_2_ fixation pathway, in part due to the lack of the required enzyme, phosphoenolpyruvate (PEP) carboxylase, which appears to be missing in these populations. The pathways responsible for CO_2_ fixation in these phylogenetic groups are still the subject of considerable research (Huber et al., 2006, 2008; Jahn et al., 2007; Ramos-Vera et al., 2009, 2011; Berg et al., 2010a,b), and numerous members of the Desulfurococcales and Thermoproteales cannot grow solely on CO_2_ and require complex sources of C for growth (Huber et al., 2006; Boyd et al., 2007; Macur et al., 2013). No Type 1 *4hbd* genes were present in the NAG1 population (Geoarchaeota) from OSP_8, or the *Caldivirga/Vulcanisaeta*-like (T1) organisms in any of these sites (Table 3). Consequently, if these organisms are capable of fixing CO_2_, it is likely occurring via a different mechanism.

## Discussion

Prior to the current study, the structure and function of hyper-thermophilic microbial communities in YNP has been inferred primarily from results of PCR using universal bacterial or archaeal primers, and inferred physiology from cultured relatives. Moreover, it has been difficult to gain any definitive information regarding the relative importance of bacteria versus archaea in high-temperature systems of YNP. The sites described here were chosen to represent several common chemotrophic community types in YNP, and included elemental sulfur systems ranging in pH from 2.5 to 6.5, as well as acidic Fe-oxide mats. Random shotgun sequencing (i.e., metagenomics) showed that these sites were dominated by archaeal populations, with the exception of *Washburn Springs*. Although other sites contained evidence of subdominant bacterial populations, sediment from WS_18 (pH = 6.4, T = 80°C) contained a significant number of sequences corresponding to *Sulfurihydrogenibium* (Aquificales), Thermodesulfobacteria and *Dictyoglomus*-like organisms. Given the circumneutral pH and the high-sulfide concentrations at WS_18, one would expect *Sulfurihydrogenibium* rather than *Thermocrinis* (higher pH) or *Hydrogenobaculum* (lower-pH) (Takacs-Vesbach et al., 2013).

The distribution of different archaeal populations as a function of environmental factors was consistent with the major habitat types identified in decision-tree format (Inskeep et al., 2013). Briefly, archaeal-dominated sites were separated based primarily on pH and the presence of dissolved sulfide and/or elemental sulfur. Temperature was not a major variable in this study since these sites were all between 72 and 85°C, and five of the seven sites were between 78 and 82°C. The abiotic consumption of oxygen by reduced sulfur species contributes to the hypoxic conditions observed in CH_1, NL_2, MG_3, JCHS_4, CIS_19, and WS_18. Consequently, six sulfidic sites were analyzed ranging in pH from 2.5 to 6.5. Our results showed that pH was a major factor controlling the distribution of Sulfolobales versus Thermoproteales and Desulfurococcales, as well as other novel archaeal groups found under limited conditions (e.g., Korarchaeota in WS_18). A combination of low pH, reduced sulfur and high-temperature severely constrained microbial community diversity and two sites with these properties were dominated by only two major Sulfolobales populations. However, the sulfidic (hypoxic) sediments at pH 6.4 (WS_18) were more diverse, and contained a significant number of *Sulfurihydrogenibium* (∼15%) and Thermodesulfobacteria-like (∼10%) sequence reads. Also, the presence of several Thermoproteales populations in WS_18 was consistent with the increased abundance of these phylotypes with increasing pH [e.g., CIS_19 (pH 4.8) and JCHS_4 (pH 6.1)]. *Washburn Springs* (WS_18) was the only habitat (out of 20 reported in the entire study, Inskeep et al., 2013) to contain a significant korarchaeotal population, and is consistent with recent studies on the distribution of korarchaeotal sequences in Kamchatka and YNP, which showed that these organisms have a limited pH range from ∼5 to 7 (Auchtung et al., 2011; Miller-Coleman et al., 2012). Although pH undoubtedly plays an important role in establishing the realized niche of thermophilic Korarchaeota (Miller-Coleman et al., 2012), sequence data suggest that this population may contain *hdrABC* gene complexes associated with sulfur oxidation via a *bd*-ubiquinol oxidase (Table 3). This population appears to have no known CO_2_ fixation pathways and may benefit from considerable levels of dissolved organic carbon (∼500 μM DOC) present in WS_18 (Table S2 in Supplementary Material, Inskeep et al., 2013).

Hydrodynamic context is also a critical modifying factor that influences the rate of equilibration with atmospheric O_2_, and is especially evident within the primary outflow channels of geothermal springs. Moderately acidic habitats (pH ∼ 3–3.5) containing Fe(II) and oxygen (i.e., OSP_8 and 14) showed an increase in archaeal diversity relative to the lower-pH habitats containing reduced sulfur. The Fe-oxide mat (OSP_8) contained three to four major lineages within the Crenarchaeota [e.g., Fe-oxidizing Sulfolobales (*M. yellowstonensis*), *Acidilobus*-like, *Vulcanisaeta*-like], and several undescribed lineages within the Thaumarchaeota, Euryarchaeota, and newly proposed Geoarchaeota (Kozubal et al., 2013). The different types of archaea and the corresponding diversity of heme Cu oxidases found in Fe mats (e.g., OSP_8, Table 3) is consistent with the fact that these are the most oxic environments included in the study. The Fe-mat also contained members of the Aquificales (*Hydrogenobaculum*-like), but these bacteria were more pronounced in filamentous “streamer” communities (site OSP_14; Takacs-Vesbach et al., 2013).

Archaea are adapted to numerous extreme environments and their respective functional attributes are equally diverse. The TIGRFAMs identified in the current study significantly expand the diversity of proteins reported from metagenome sequence currently in public databases. This is due primarily to the abundant and diverse archaea distributed across these sites and the fact that few metagenomes from high-temperature systems have been reported. The presence of different functional genes among high-temperature chemotrophic communities is defined by the distribution of predominant archaeal phylotypes and provides a foundation for understanding metabolic linkages to environmental constituents such as O_2_, S, and Fe, as well as the evolutionary history of these phyla. Likewise, the lack of genes known to code for the oxidation of ammonium (*amoA*) and/or methane (*pmoA*) suggests that these reactions, although exergonic, do not support the metabolism of dominant populations in these sites. The archaeal sites sampled here do not contain significant numbers of methanogens with the exception of WS_18, where the higher pH (6.4), dissolved CO_2_ and dissolved H_2_ (Table 1) appear to support subdominant populations (<1% of total sequences) related to Methanococci and/or Methanosarcinales. Other poorly characterized archaea identified across these high-temperature systems included members of the Nanoarchaeota, Euryarchaeota, and novel phylum-level lineages (e.g., Geoarchaeota from OSP_8). Moreover, the presence of viral sequence in the community metagenomes as well as the identification of unique CRISPR regions in numerous archaeal phylotypes provides genomic evidence that new viruses have yet to be identified and characterized in these habitats. Although there are numerous high-temperature habitats yet to be studied, the sites included here provide an excellent foundation for understanding both phylogenetic and functional variation within the archaea as a function of major geochemical parameters including pH, reduced sulfur, dissolved oxygen, and ferrous Fe.

## Materials and Methods

### Site selection, sample collection, and processing

Seven high-temperature sediment and/or mat samples rich in archaea (Figure 1) were sampled from geothermal environments in 2007–2008. The sites were chosen to obtain a range in pH across hypoxic sulfur sediments (2.5–6.4), as well as to contrast reduced sulfur environments with oxic flow channels containing Fe(III)-oxides. The research sites chosen for study have been the subject of significant prior characterization and include: *Crater Hills* (CH_1, *Alice Springs*), *Nymph Lake* (NL_2), *Monarch Geyser* (MG_3), *Cistern Spring* (CIS_19), *Joseph’s Coat Hot Spring* (JCHS_4, also known as “JC3” and *Scorodite Spring*), *Washburn Springs* (WS_18), and *One Hundred Springs Plain* (OSP_8). Each microbial community and associated solid phase was sampled aseptically, stored in 50 mL sterile “falcon” tubes on dry ice, and transported to a −80°C freezer (MSU) until DNA extraction.

Parallel samples of the bulk aqueous phase (<0.2 μm) associated with each microbial community were obtained simultaneously and analyzed using a combination of field and laboratory methods. As described in more detail in other reports (Inskeep et al., 2004; Macur et al., 2004), pH, temperature, and other redox sensitive species [Fe^II^/Fe^III^; As^III^/As^V^; total dissolved sulfide (DS); dissolved O_2_ (DO)] were determined using field methods. Major cations and other trace elements (i.e., Na, K, Ca, Mg, Fe, Al, Mn, Cu, Sr, Ba, Li, Zn, Cu, Pb, Si, B, P, As, Sb, S, Se) were determined using inductively coupled plasma (ICP) spectrometry, and major anions (F, Cl, SO_4_, S_2_O_3_, AsO_4_, PO_4_, NO_3_) were determined using ion chromatography (Dionex, Sunnyvale, CA, USA). Ammonium concentrations were determined using colorimetry (autoanalyzer). Dissolved gases (CO_2_, H_2_, and CH_4_) were determined using closed head-space gas chromatography (Inskeep et al., 2005) of sealed serum-bottle samples obtained in the field. The majority of these sites have been sampled many times with excellent replication (Langner et al., 2001; Rice et al., 2001; Inskeep et al., 2005, 2010; Young et al., 2005; Kozubal et al., 2012, 2013). The location and primary physicochemical characteristics obtained during sampling are provided here (Table 1); additional geochemical data are included in supplemental information (see Table S2 in Supplemental Material, Inskeep et al., 2013).

### DNA extraction and library construction

Although a standard DNA extraction protocol (Inskeep et al., 2013) was attempted for all samples, several of the archaeal-dominated sediment samples required extraction kits (MoBio) to obtain sufficient DNA for analysis. Our main emphasis was to obtain representative, unbiased community DNA as template for construction of small insert libraries. Small insert (puc13) libraries were constructed and transformed, then sequenced (Sanger ∼ 800 bp reads) to generate ∼35–50 Mb per site, for a total sequence for these sites of ∼250 Mb. Sites NL_2, JCHS_4, and MG_3 also received a half-plate of 454 pyrosequencing. For consistency, this manuscript focuses on the Sanger data across each of the seven sites; pyrosequence data did not result in assemblies containing significantly greater non-redundant sequence, although it did improve the coverage of dominant community members.

### Analysis of individual sequence reads

Analysis of individual sequence reads using MEGAN assignments from “blastx” results and G + C content distribution provided a quick and useful phylogenetic summary of predominant community members in each site (Figure 2), and indicated where to expect major assemblies. Whole genome-level comparative analysis was accomplished using fragment (read) recruitment of environmental sequence data to reference microbial genomes (Rusch et al., 2007). At the time of writing, the database contained microbial genomes for ∼1,500 bacteria and 100 archaea. Currently, only a handful of microbial genomes served as appropriate references for the indigenous organisms within these chemotrophic communities, consequently, many of the assignments were given at family or domain level.

### Analysis of predominant sequence assemblies

Random shotgun DNA sequence (∼35–50 Mbp Sanger per site) was obtained and assembled as described in this issue (Inskeep et al., 2013). Assembled metagenome sequence data was analyzed using PCA of NWF (Teeling et al., 2004; Inskeep et al., 2010) calculated for all contigs/scaffolds greater than 3–4 kb. The sequence clusters were also viewed with a simultaneous phylogenetic classification based on the (APIS; Badger et al., 2006), or a “blast”-based classification (Rusch et al., 2007). Briefly, APIS is a system for automatic creation and summarizing of phylogenetic trees for each protein encoded by a genome or metagenomic dataset.

### CRISPR analysis

CRISPRs were identified using the CRISPRFinder software (Grissa et al., 2007). DR were counted and individual repeats with less than 10 instances in the assembly were excluded from further analysis. The CRISPR spacer and DR were searched (NCBI BLASTN with –e 100 –U T –F “m L” –b 10000; Altschul et al., 1990) against the scaffolds and HSPs with up to three or fewer mismatches were identified. Scaffolds identified by the CRISPR spacers were searched against NRAA (BLASTX –e 1e-4 –U T –F “m L”) to determine their similarity to known viruses. Spacer identified scaffolds were also searched against themselves (BLASTN –U T –F “m L” –X 150 –q –5 –r 4).

### TIGRFAM protein family abundance in assembled metagenome sequence

Assembled sequence from each of the archaeal sites was annotated as described in Inskeep et al. (2010) and predicted proteins from the scaffolds were assigned TIGRFAM protein families (Selengut et al., 2007) using HMMER 3 (Eddy, 2011) with E-value cutoff of 1e-6. PCA and statistical analysis of site group differences was performed using the STAMP v2.0 software (Parks and Beiko, 2010). The White’s non-parametric *t*-test and ANOVA tests were used to test for differences between two site groups and multiple site groups respectively. Two-way clustering was done using row-standardized (across sites) average TIGRFAM category abundance data using the Euclidean distance metric and complete-linkage hierarchical clustering in MeV 4.8 (Saeed et al., 2003) software. Other details are as described above (Inskeep et al., 2013).

### Functional analysis of archaea in YNP

The assembled sequence data was screened for specific functional genes corresponding to known and/or putative pathways in carbon metabolism and electron (energy) transfer. We were specifically interested in assessing metabolic potential for chemolithoautotrophy (CO_2_ fixation and electron transfer) in high-temperature geothermal systems. Query DNA sequences known to code for proteins important in the oxidation of reduced chemical constituents or the reduction of a terminal acceptor were used to search the environmental sequence data. Environmental sequence fragments exhibiting homology (E-values <10^−10^) to query sequences (Table S3 in Supplementary Material, Inskeep et al., 2013) were then reanalyzed using “blastn,” and carefully assessed individually using phylogenetic analysis of deduced protein sequences against known relatives, as well as fragment length relative to query length. False positives were eliminated by this screening process and included (i) sequences matching the correct protein family, but not the exact query sequence (e.g., Mo-pterin oxidoreductases versus a specific protein within this family), (ii) sequences that matched a query gene due to homologous regions, but were clearly associated with a gene cluster of different function, and (iii) sequences that returned mis-annotated “blastn” relatives. It is also possible that our inventory of metabolic potential has missed sequences related to a specific query gene. For example, some homologous genes found in the metagenome data were of insufficient length relative to known query sequences to make a definitive assignment. Clearly, the metagenomes obtained here do not represent complete sequence for all subdominant populations in these sites.

### Sequence availability

All annotated metagenome sequence assemblies (Celera/PGA) discussed in the current manuscript are available through the DOE-JGI IMG/M (Markowitz et al., 2012) website (http://img.jgi.doe.gov/m) under IMG taxon OID numbers as follows: YNPSite01 (2022920009/2014031002), Site02 (2022920014/2015219001, 2016842002), Site03 (2022920002/2014031003, 20162001), Site19 (2022920017/2015219000), Site04 (2022920008/2013843003), Site18 (2022920019/2016842004), Site08 (2022920005/2013515001) and Site14 (2022920007/2013954001). Scaffold ID numbers are preserved in the annotated Celera sequence files, and serve as an appropriate mechanism of referencing assembled sequence data.

## Conflict of Interest Statement

The authors declare that the research was conducted in the absence of any commercial or financial relationships that could be construed as a potential conflict of interest.

## Supplementary Material

The Supplementary Material for this article can be found online at http://www.frontiersin.org/Microbial_Physiology_and_Metabolism/10.3389/fmicb.2013.00095/abstract

Supplementary Table S1 **Summary of 16S rRNA gene sequences observed in assembled metagenome sequence data from high-temperature, archaeal-dominated chemotrophic sites in Yellowstone National Park (also, see Figure 4 for phylogenetic tree)**.

Supplementary Table S2 **Description of predominant sequence assemblies in archaeal-dominated sites, including largest scaffold (kbp), number of scaffolds in cluster, total consensus sequence (Mbp), average G + C content (%), and closest cultured relative of the 16S rRNA gene found within the assembled data**.

Supplementary Table S3 **Distribution of direct repeats (DR) in archaeal-dominated sites**.

Supplementary Table S4 **Survey of single-copy genes corresponding to the predominant archaeal populations present in high-temperature geothermal microbial communities of YNP**.
