# Supplementary material for: Phylogenetic and Functional Analysis of Metagenome Sequence from High-Temperature Archaeal Habitats Demonstrate Linkages between Metabolic Potential and Geochemistry
Source: Front Microbiol. 2013 May 15;4:95. doi: 10.3389/fmicb.2013.00095 (PMC3654217; doi:10.3389/fmicb.2013.00095)
Supplement: Supplementary Table S1 — Summary of 16S rRNA gene sequences observed in assembled metagenome sequence data from high-temperature, archaeal-dominated chemotrophic sites in Yellowstone National Park (also, see Figure 4 for phylogenetic tree). [file 41699_Inskeep_DataSheet1.PDF]

Table 1. Sample location, total dissolved (< 0.2 um) geochemical parameters[1], and predominant solid phases associated with high-temperature, archaeal-dominated microbial communities in Yellowstone National Park (YNP).

Location	T	pH	I	SO_4	NH_4	DIC	DS	DO	S_2O_3	As	Fe	CH_4	H_2	Solid Phases[2]	YNP Thermal Inventory	Coordinates
	°C		-- mM --				---------------- µM ---------------						nM			
**Crater Hills (CH_1)**	76	2.5	18	8.7	7.2	1.4	2	< 3	< 3	2	230	0.34	67	S^0, SiO_2	‘Alice Spring‘ CHANN041	44° 39' 11.823" N. Lat 110° 29' 5.067" W. Lon
**Nymph Lake (NL_2)**	88	~ 4[3]	17	1.3	0.23	0.6	3	< 3	< 3	9	1.5	<0.01	84	S^0, SiO_2	West of Nymph Lake	44° 45’ .8.354” N. Lat 110° 43’ 31.413” W. Lon
**100 Springs Plain (OSP_8)**	72	3.3	14	1.1	0.09	0.2	< 1	30	< 3	28	36	<0.01	34	$Fe(AsO_4)(OH)_x$	NHSP115	44° 43’ 58.953” N. Lat 110° 42’ 32.374” W. Lon
**Monarch Geyser (MG_3)**	80	4.0[3]	16	1.3	0.28	1.4	25	< 3	55	19	3.6	0.61	375	S^0, SiO_2	NBB218	44° 43’ 27.453” N. Lat 110° 42’ 20.207” W. Lon
**Cistern Spring (CIS_19)**	78	4.4[3]	11	0.7	0.30	1.2	20	< 3	200	19	0.2	0.30	460	S^0, SiO_2, $KAl_3SO_4(OH)_6$	NBB201	44° 43’ 23.092” N. Lat 110° 42’ 14.489” W. Lon
**Joseph’s Coat Hot Springs (JCHS_4)**	80	6.1	23	3.8	8.4	0.2	20	< 3	700	140	1.6	0.19	65	Sb_2S_3, FeS_2, As_2S_3, S^0, SiO_2	Scorodite Spring JCS083	44° 44' 20.984” N. Lat 110° 19' 28.179" W. Lon
**Washburn Springs (WS_18)**	76	6.4	42	16.7	24	5.5	160	< 3	< 3	1.6	1.1	14.9	450	S^0, FeS_2, SiO_2	Washburn Spring	44° 45' 53.62” N. Lat 110° 25' 49.26 " W. Lon

[1] I = ionic strength calculated from aqueous geochemical modeling at sample temperature; DIC = dissolved inorganic C; DS = dissolved sulfide; $S_2O_3{}^{2-}$ = thiosulfate; DO = dissolved oxygen.

[2] predominant solid phases determined using scanning electron microscopy (FE-SEM) coupled with energy dispersive analysis of x-rays (EDAX) and x-ray diffraction (XRD).

[3] pH values at these sites have been noted to vary (MG_3; pH ~ 4-4.5; CIS_19; pH ~ 4.4-4.9). Also see USGS reports (e.g., Ball et al., 1999; 2002; McCleskey et al., 2005)

Table 2. Scaffolds with similarity to known viruses. The source of the assembled reads, %G+C, scaffold length, and the number of ambiguous bases (N's) in the scaffolds are shown. Scaffolds showing nucleotide sequence similarity to each other are identified by the 'Similarity Groups (A, B), where the similar scaffolds are listed. The best match to known viral genomes (BLASTX to the NRAA database) is shown along with the length of that genome. Sequence from eight of these scaffolds contained matches to CRISPR spacer regions (3 mismatches allowed).

Viral Scaffold Characteristics					CRISPR Information		Similarity Information			
**Sample**	**Scaffold ID[1]**	**%G+C**	**Scaffold Length**	**Number of N's**	**Spacer Regions**	**CRISPR Site**	**Similarity Group**	**Matching Scaffolds**	**Matching Viral Genome**	**Genome Length**
**YNP_02**	scf_6656549	56.3	18010	0	1	2	A	scf_5653343 scf_6606346	Thermoproteus tenax spherical virus 1	20993
**YNP_03**	scf_6648705	41.4	12461	20	4	3*	B	scf_5653210	Sulfolobus islandicus rod-shaped virus 1	32308
**YNP_03**	scf_6649099	38.2	34046	0	0	-	-		Acidianus filamentous virus 9	41172
**YNP_03**	scf_6649105	40.5	16038	0	1	3	-		Sulfolobus islandicus filamentous virus	40900
**YNP_04**	scf_6606346	56.5	15839	0	0	-	A	scf_5653343 scf_6656549	Thermoproteus tenax spherical virus 1	20993
**YNP_04**	scf_6606202	58.5	27771	653	9	4	-		Acidianus rod-shaped virus 1	24655
**YNP_14**	scf_6615532	27.6	34310	668	5	14*	-		Sulfolobus islandicus rod-shaped virus 1	32308
**YNP_19**	scf_5653343	56.1	18048	20	1	19	A	scf_6656549 scf_6606346	Thermoproteus tenax spherical virus 1	20993
**YNP_19**	scf_5653210	42.7	13852	567	5	19	B	scf_6648705	Sulfolobus islandicus rod-shaped virus 2	35450
**YNP_19**	scf_5653402	50	16801	0	1	19	-		Acidianus filamentous virus 2	31787

[1] complete scaffold ID (_ = 111868) retained as an identifier in sequence assemblies deposited with IMG/M.

Table 3. Summary of key metabolic genes identified in metagenome sequence of the predominant archaeal populations[1] present across sites exhibiting a wide range in pH, dissolved sulfide, and dissolved oxygen (see Table 1).

Process/Pathway	Marker Genes[1]	Crater Hills (CH)	Nymph Lake (NL)	100 Spr. Plain (OSP)	Monarch Gysr. (MG)	Cistern Spr. (CIS)	J. Coat Hot Spr. (JCHS)	Washburn Spr. (WS)
CO_2 fixation								
4-hydroxybutyryl-CoA dehydratase	Type1 *4hbd*	**S1, S2**	**S1**	**My**	**A[2], S**	**A[2], S1, T2**	**T2**	
Acetyl-CoA/Propionyl-CoA carboxylase	*accA*	**S1, S2**	**S1**	**My**		**S1, S2**	**S1**	
Electron Donors-Acceptors								
Hydrogen Oxidation (Group 1 Ni-Fe)	*hynSL*	**S1, S2**	**S1**		**S**	**S**	**S, T2**	**T2, T3**
Thiosulfate Oxidation	*tqoA,B*	**S1, S2**	**S1**	**My, S**	**S**	**S**	**S**	
Sulfur Oxidation	*sor*	**S2**						
Elemental S Oxidation	*hdrABC*	**S1, S2**	**S1**	**My, S**	**S**	**S**	**S**	**K**
Sulfide Oxidation	*sqr*	**S1, S2**	**S1**	**My, S**		**S**	**S**	**Sfy**
Reduced S Oxidation (S-transferase)	*rdh*	**S1, S2**	**S1**	**NAG1, My, T1, Thaum**	**T1**	**T1**	**T1, S**	**Sfy**
Sulfite Oxidation (Mo-pterin)	*soxBC*	**S1, S2**	**S1**	**A, T1, T2, S**	**A, T1, T2, S**	**A, T1, T2, S**	**A, T1, T2, S**	**Sfy, T2**
Oxygen Reduction, Heme Cu Oxidases[3]	*e.g. doxB, foxA*	**S1 doxB S2 doxB**	**S1 doxB**	**NAG1-soxM, My-foxA/doxB, S-soxB/soxM**			**S-doxB, S-soxB**	**Sfy-coxA**
Oxygen Reduction, cytochrome *bd*-ubiquinol type	*cytAA'*	**S2**	**S1**	**NAG1, A, T1**	**A, T1**	**A, T1, T2**	**A, T1, T2**	**Sfy, K, T2, T3**
Dissimilatory Reduction of Sulfur[4] (DMSO Mo-pterin family)	*psrA/sreA*[4]	**S1, S2**	**S1**	**My**		**T2[4]**	**T2[4]**	**Sfy[4],T2[4]**
Dissimilatory Reduction of Sulfur (NADPH Elemental S reductase)	*nsr*	**S1, S2**	**S1**	**My, A, T1**	**A, T1**	**A, T1, S2**	**A, T1, T2**	
Dissimilatory Reduction of Sulfate	*dsrAB*			**T1, T2**	**T1, T2**	**T1, T2**	**T1, T2**	**T2, Tds**
Reduction of Nitric oxide	*norB*			**T1**		**T1, T2**	**T1, T2**	**T2**
Detoxification-Stress Response	*arsB*	**S1, S2**	**S1**	**NAG1, My, A, T1**	**A, T2**	**A, T1, T2, S**	**A, T1, T2**	**Sfy, K, T2, T3**
	arsC			**NAG1**				**Shy, Dt**
	merA	**S2**	**S1**	**NAG1, S**		**S**	**S**	**T2**
	sodA	**S1, S2**	**S1**	**NAG1, My, A, T1, S**	**A, T1, S**	**A, T1, T2, S**	**A, T1, T2, S**	**Sfy,T2, Mth, Tds**

[1] Population types: S1 and S2 =Sulfolobales Types 1 and 2, respectively; My =.M. yellowstonensis; A = Desulfurococcales (*Acidilobus* sp.); T1, T2, T3 = Thermoproteales Types 1, 2 and 3, respectively; NAG1 = 'Novel Archaea Group 1 (Candidate phylum Geoarchaeota; Kozubal et al., 2012b)'; K = Korarchaeota; Sfy = *Sulfurihydrogenibium*-like; Dt = *Dictyoglomus*-like; Mth = Methanosarcinales; Tds = Thermodesulfobacteria.

[2] 'high confidence' sequence matches to marker genes that code for proteins with high specificity for possible pathway. Query sequences used to search for specific functional genes given in Table S3 (Inskeep et al., 2013). Not present in these sites = *amoA, nifH, mcrA, nirK, nirS, nosZ.*

[3] specific heme Cu oxidases (sub-unit 1) are listed for each population type

[4] uncharacterized DMSO-Mo-pterins belonging to the Thermoproteales (T2) and *Sulfurihydrogenibium* (Sfy) populations are listed here.

Table S1. Summary of 16S rRNA gene sequences observed in assembled metagenome sequence data from high-temperature, archaeal-dominated chemotrophic sites in YNP (also see Figure 4 for phylogenetic tree).

Location (Abbrev Site #)	NCBI Closest Cultivated Relative[1]	Fragment Length (bp)	Identity (%)	E-value	Score	NCBI No.	Celera Scaffold ID
**Crater Hills (CH_1)**	*Sulfolobus tokodaii str. 7*	1501	91.5	0	2050	NC_003106	scf1118683610939
	Stygiolobus azoricus	1495	99.6	0	2582	X90480	scf1118683610477
**Nymph Lake (NL_2)**	*Sulfolobus tokodaii str. 7*	1500	91.6	0	2056	NC_003106	scf1118686656987
	Nanoarchaeum equitans Kin4-M	1405	81.8	0	1123	NC_005213	scf1118686656623
**One Hundred Spring Plain (OSP_8) Fe-mat**	*Thermofilum pendens* Hrk 5	[2]1492	82.2	0	1247	NC_008698	scf1118686596894
	Acidilobus saccharovorans 345-15	944	97.2	0	1594	NC_014374	scf1118686595095
	Hydrogenobaculum sp. Y04AAS1	676	98.2	0	1182	NC_011126	scf1118686594822
	Vulcanisaeta distributa DSM 14429	657	98.5	0	1157	NC_014537	scf1118686595630s
	Nanoarchaeum equitans Kin4-M	463	83.5	5.00E-113	418	NC_005213	cf1118686595700
**One Hundred Spring Plain (OSP_14) Fe-streamer**	*Metallosphaera yellowstonensis* MK1	1507	99	0	2952	-	scf1118686615930s
	Hydrogenobaculum sp. Y04AAS1	1436	99.1	0	2580	NC_011126	cf1118686615567
	Nanoarchaeum equitans Kin4-M	707	82.5	3.00E-162	592	NC_005213	scf1118686616079
**Monarch Geyser (MG_3)**	*Acidilobus saccharovorans* 345-15	1498	96.7	0	2492	NC_014374	scf1118686649184
	Caldivirga maquilingensis IC-167	616	97.9	0	1059	NC_009954	scf1118686646012
**Cistern Spring (CIS_19)**	*Acidilobus saccharovorans* 345-15	1498	96.7	0	2508	NC_014374	scf1118685653408
	Vulcanisaeta distributa DSM 14429	1060	98.1	0	1845	NC_014537	scf1118685652594
	Caldivirga maquilingensis IC-167	443	96.4	0	730	NC_009954	scf1118685653008
	Stygiolobus azoricus	424	99.8	0	769	X90480	scf1118685652166
**Joseph's Coat Hot Springs (JCHS_4)**	*Acidilobus saccharovorans* 345-15	1498	96.6	0	2481	NC_014374	scf1118686607546s
	Thermoproteus neutrophilus V24Sta	1103	97.2	0	1770	NC_010525	cf1118686607488
	Caldivirga maquilingensis IC-167	616	97.9	0	1059	NC_009954	scf1118686606021
	Vulcanisaeta distributa DSM 14429	609	97.1	0	1024	NC_014537	scf1118686607276
	Nanoarchaeum equitans Kin4-M	455	83.8	6.00E-113	420	X90480	scf1118686606049
**Washburn Springs (WS_18)**	*Sulfurihydrogenibium* sp. Y03AOP1	1514	98.8	0	2695		scf1118685639305
	Korarchaeota SRI-306	1334	98.9	0	2379		scf1118685637308
	Staphylothermus sp. 1633	1345	93.2	0	1954		scf1118685637284
	Dictyoglomus turgidum DSM 6724	990	97.5	0	1681		scf1118685639135
	Fervidobacterium sp. YNP	714	99.0	0	1279		deg1118685632001
	Pyrobaculum sp. CBA1503	481	98.7	0	852		scf1118685641534

[1] Although closest cultivated relatives are listed here, these specific '*genus species*' must not be over-interpreted as strict phylogenetic assignments (see Figure 4 for phylogenetic tree).

[2] This sequence belongs to the 'Novel Archaeal Group 1' population (Candidate phylum Geoarchaeota; Kozubal et al., 2012b).

Table S2. Predominant sequence types (clusters) organized by phylogenetic assignment. Cluster parameters include largest scaffold (kbp), number of scaffolds in cluster, total consensus sequence (Mbp), average G+C content (%), and closest cultured relative of the 16S rRNA gene found within each cluster.

Cluster Assignment	Site	Largest scaffold (kb)	# scaffolds	Total Sequence (Mb)[1]	Average G+C %	Closest cultured relative (16S rRNA gene length; % nucleotide identity)
**Sulfolobales Type 1**	**CH_1**	515	14	**1.52**	51.5	***Sulfolobus* sp. (1497, 91)**
**Sulfolobales Type 1**	**NL_2**	755	13	**1.55**	51.5	***Sulfolobus* sp. (1497, 92)**
**Sulfolobales Type 1**	**CIS_19**	22	67	**0.5**	52	**-**
**Sulfolobales Type 2**	**CH_1**	258	13	**1.39**	36	***Stygiolobus* sp. (1495, 99)**
**Sulfolobales Type 2**	**CIS_19**	43	161	**1.8**	39	**-**
**M. yellowstonensis-like**	**OSP_8**	25	175	**1.2**	48	**-**
**M. yellowstonensis-like**	**OSP_14**	108	72	**1.94**	48.6	***M. yellowstonensis* (1497, 98)**
**Desulfurococcales Type 1**	**OSP_8**	380	42	**1.4**	59	***Acidilobus* sp. (944, 97)**
**Desulfurococcales Type 1**	**MG_3**	471	35	**1.5**	59	***Acidilobus* sp. (1497, 97)**
**Desulfurococcales Type 1**	**CIS_19**	346	18	**1.4**	59	***Acidilobus sp. (1498, 97)***
**Desulfurococcales Type 1**	**JCHS_4**	388	13	**1.5**	59	***Acidilobus sp. (1498, 97)***
**Thermoproteales Type 1**	**OSP_8**	34	241	**2.1**	46	**-**
**Thermoproteales Type 1**	**MG_3**	50	381	**3.0**	48	***Caldivirga sp. (616, 98)***
**Thermoproteales Type 1**	**CIS_19**	37	312	**3.2**	47	***Caldivirga sp. (450, 96)***
**Thermoproteales Type 1**	**JCHS_4**	77	344	**3.9**	47	***Caldivirga sp. (824, 98%)***
**Thermoproteales Type 2**	**CIS_19**	70	110	**1.8**	61	**-**
**Thermoproteales Type 2**	**JCHS_4**	170	212	**3.1**	60	***Thermoproteus sp. (1103, 97)***
**Novel Archaea Group I**	**OSP_8**	1,357	8	**1.7**	32.5	***Thermophilum (1492, 82)***[2]
**Nanoarchaeota**	**OSP_8**	13	16	**0.1**	27	***Nanoarchaeum sp. (463, 84)***
**Nanoarchaeota**	**OSP_14**	11	21	**0.1**	27	***Nanoarchaeum sp. (700, 82)***

[1] total consensus sequence for several of these population types is deposited separately on IMG/M (Markowitz et al., 2012) as well as the UCSC archaeal browser. Table S4 provides a partial summary of single-copy gene analysis for several population types.

[2] Candidate phylum Geoarchaeota; Kozubal et al., 2012b.

Table S3. Distribution of direct repeats (DR) in archaeal-dominated sites. Counts represent the number of near perfect matches to the DRs found in the metagenome assemblies (1 mismatch allowed). The total HSP count provides the sum of HSPs for a particular DR found in the archaeal samples, whereas the maximum number of HSPs represents the highest count for any single sample across all 20 Yellowstone samples. The fraction of DR sequence for each site is calculated based on the total DRs observed across these sites; the values in a row should sum to 1.

Direct Repeat	Genus	Total HSP Count	Max Count	CH_1	NL_2	MG_3	JCHS_4	OSP_8	WS_18	CIS_19
CTTTCAATCCCTTTTGGGATGCAAC	**Sulfolobus**	40	34	0.85	0.00	0.00	0.00	0.03	0.00	0.13
CTTTCAATTCCATTGAGGATTAAC	**Sulfolobus**	71	41	0.58	0.00	0.04	0.00	0.14	0.00	0.24
GTAACAACTGAAAGAAACTAAAAC	**Sulfolobus**	660	431	0.26	0.65	0.04	0.02	0.00	0.00	0.02
GTAACAACTGAAAGAAACTAAAA	**Sulfolobus**	18	13	0.22	0.72	0.06	0.00	0.00	0.00	0.00
CTTTCAGTTCTTCCTTATTTCGAC	**Sulfolobus**	49	36	0.04	0.02	0.04	0.00	0.16	0.00	0.73
CTTTCAATATTCTATTGAAATCAAC	**Sulfolobus**	18	8	0.00	0.06	0.22	0.28	0.00	0.00	0.44
GTTGCAACGCAAAGAAACTAAAAC	**Sulfolobus**	107	83	0.00	0.00	0.78	0.14	0.04	0.00	0.05
GTTTCAAATCCTTATAGGTATTCTACAAAC	**Sulfolobus**	18	72	0.00	0.00	0.28	0.00	0.72	0.00	0.00
CTTTCAATTCCATTAAGGATTAAC	**Sulfolobus**	8	7	0.00	0.00	0.13	0.00	0.00	0.00	0.88
CCTTCTACTCCCTTTAGGGAGATTC	**Sulfolobus**	41	23	0.00	0.00	0.07	0.56	0.10	0.00	0.27
CTTTCAATTCCTTTGAGGATGAAAC	**Sulfolobus**	37	83	0.00	0.00	0.00	0.00	1.00	0.00	0.00
CTTTCAATTCTATCCAAGATCAAC	**Sulfolobus**	21	31	0.00	0.00	0.00	0.00	1.00	0.00	0.00
CTTTCAATTCTTTCTGAGTTGCATC	**Thermofilum**	47	39	0.00	0.00	0.00	0.17	0.00	0.83	0.00
CTTAAACTCAGAAGAGGATTGAAAG	**Thermofilum**	168	168	0.00	0.00	0.00	0.00	1.00	0.00	0.00
CCTTCAATTCTCCTTTGAGATCTTC	**Caldivirga**	35	17	0.00	0.03	0.49	0.20	0.09	0.00	0.20
CCTTCAATTCTCTTTTGAGATCTTC	**Caldivirga**	68	33	0.00	0.01	0.49	0.19	0.13	0.01	0.16
CCTTCAATTCTCCTTTGAGATCCTC	**Caldivirga**	27	18	0.00	0.00	0.67	0.04	0.15	0.00	0.15
CCTTCTACTCCCTTTAGGGAGTTTC	**Caldivirga**	29	15	0.00	0.00	0.52	0.17	0.07	0.00	0.24
CCTTCAATTCTCTTTTGAGATCCTC	**Caldivirga**	27	12	0.00	0.00	0.44	0.07	0.11	0.00	0.37
GAATCTAAAACAGAAGAATTGAAGC	**Caldivirga**	45	16	0.00	0.00	0.36	0.18	0.13	0.00	0.33
CTTTCAATACTCTATTAGAGATGC	**Caldivirga**	24	17	0.00	0.00	0.08	0.71	0.04	0.00	0.17
GTAGAATCTCAAGAAGGGTTGAAAC	**Pyrobaculum**	58	26	0.00	0.03	0.09	0.45	0.00	0.09	0.34
CTTTCAATATTCTATTGAAATCATC	**Pyrobaculum**	41	18	0.00	0.02	0.12	0.37	0.05	0.00	0.44
GCATCTCCAATGGAGAATTGAAAC	**Pyrobaculum**	20	11	0.00	0.00	0.20	0.55	0.00	0.00	0.25
GAATCTCAAGAGAAGGATTGAAAC	**Pyrobaculum**	25	15	0.00	0.00	0.16	0.60	0.04	0.00	0.20
CTAGAATCTCAAGAAGGGTTGAAAC	**Pyrobaculum**	10	13	0.00	0.00	0.00	0.50	0.00	0.50	0.00
GCATCTCCAATAGAGAATTGAAAC	**Pyrobaculum**	11	6	0.00	0.00	0.00	0.45	0.00	0.55	0.00
CCAGAATCTCAAGAAGGGTTGAAAC	**Pyrobaculum**	6	13	0.00	0.00	0.00	0.33	0.00	0.67	0.00
CTTTCAATTCTCTATTTGAGATGC	**Pyrobaculum**	33	24	0.00	0.00	0.00	0.27	0.00	0.73	0.00

Supplemental Table 4. Survey of single-copy genes corresponding to the predominant archaeal populations present in high-temperature geothermal microbial communities of YNP.

Cluster Type	Site	Archaeal RNA Polymerase subunits (excluding RpoG & Rpo13)												
		RpoA'	RpoA"	RpoB	RpoD	RpoL	RpoE'	RpoE"	RpoF	RpoH	RpoK	RpoM/TFS	RpoN	RpoP
**Sulfolobales I**	**CH_1**	1	1	1	1	1	1	1	1	1	1	1	1	1
**Sulfolobales II**	**CH_1**	0	0	0	0	1	0	1	1	1	1	1	0	0
**Sulfolobales I**	**NL_2**	1	1	2	1	1	1	1	1	1	1	1	1	1
**Sulfolobales I**	**CIS_19**	1	1	0	1	1	0	0	0	0	1	1	1	0
**Sulfolobales II**	**CIS_19**	1	1	0	0	0	1	1	1	0	0	3	1	1
M. yellowstonensis	**OSP_8**	0	0	0	0	0	0	0	0	0	1	1	0	0
M. yellowstonensis	**OSP_14**	0	1	1	0	1	0	0	1	1	1	1	1	1
**Desulfurococcales**	**MG_3**	1	1	1	1	1	1	1	1	1	1	1	1	1
**Desulfurococcales**	**JCHS_4**	1	1	1	1	1	1	1	1	1	1	2	1	1
**Desulfurococcales**	**OSP_8**	2	2	1	1	1	1	1	1	1	1	2	1	0
**Desulfurococcales**	**CIS_19**	1	1	1	1	1	1	1	1	1	1	2	1	1
**Thermoproteales I**	**MG_3**	1	2	2	1	2	1	0	2	1	2	4	2	2
**Thermoproteales I**	**JCHS_4**	1	1	1	1	1	0	1	1	1	1	4	1	2
**Thermoproteales I**	**OSP_8**	0	0	1	2	1	2	1	1	0	2	2	1	1
**Thermoproteales I**	**CIS_19**	1	1	1	1	2	0	1	2	1	2	3	1	2
**Thermoproteales II**	**JCHS_4**	2	2	3	2	2	2	2	2	2	1	5	2	1
**Thermoproteales II**	**CIS_19**	1	1	1	1	1	1	1	1	1	1	2	1	0
**Geoarchaeota (NAG1)***	**OSP_8**	1	1	1	1	1	1	1	1	1	1	1	1	1
**Nanoarchaeota**	**OSP_8**	1	1	1	0	0	1	1	0	1	0	0	0	0

*All protein hits (except 1 TFE hit) are on the same scaffold: scf1118686596894

Table S4 (page 2).

Cluster Type	Site	Transcription Factors							RNAse P			
		NusA	NusG	TBP	TFB	TFE	TFIIH	TIP49	pop5	rpp21	rpp29	rpp30
Sulfolobales I	CH_1	1	1	1	1	1	1	1	1	1	1	1
Sulfolobales II	CH_1	0	1	1	2	1	1	1	1	1	0	1
Sulfolobales I	NL_2	1	1	1	2	1	1	1	1	1	1	1
Sulfolobales I	CIS_19	0	0	1	0	1	0	0	1	0	0	1
Sulfolobales II	CIS_19	1	1	1	2	1	1	1	1	0	1	1
M. yellowstonensis	OSP_8	0	1	0	2	0	0	1	1	0	0	1
M. yellowstonensis	OSP_14	1	1	1	2	1	1	0	1	1	0	1
Desulfurococcales	MG_3	1	1	1	2	1	0	2	1	0	2	1
Desulfurococcales	JCHS_4	1	1	1	2	1	0	1	1	1	1	1
Desulfurococcales	OSP_8	2	2	1	2	1	0	1	1	2	1	1
Desulfurococcales	CIS_19	1	1	1	2	1	0	1	1	1	1	1
Thermoproteales I	MG_3	1	1	1	3	2	2	2	1	0	1	0
Thermoproteales I	JCHS_4	1	2	3	6	2	2	2	1	0	1	2
Thermoproteales I	OSP_8	0	0	1	4	0	3	2	0	0	1	0
Thermoproteales I	CIS_19	1	2	2	1	1	1	2	0	0	1	1
Thermoproteales II	JCHS_4	3	1	3	6	1	3	2	1	0	2	2
Thermoproteales II	CIS_19	1	1	1	5	1	1	1	0	0	1	0
Geoarchaeota (NAG1)*	OSP_8	1	1	1	1	2	1	1	0	1	1	0
Nanoarchaeota	OSP_8	0	1	1	0	1	0	1	0	0	0	0
